# Mutation in the FUS nuclear localisation signal domain causes neurodevelopmental and systemic metabolic alterations

**DOI:** 10.1242/dmm.050200

**Published:** 2023-10-23

**Authors:** Zeinab Ali, Juan M. Godoy-Corchuelo, Aurea B. Martins-Bach, Irene Garcia-Toledo, Luis C. Fernández-Beltrán, Remya R. Nair, Shoshana Spring, Brian J. Nieman, Irene Jimenez-Coca, Rasneer S. Bains, Hamish Forrest, Jason P. Lerch, Karla L. Miller, Elizabeth M. C. Fisher, Thomas J. Cunningham, Silvia Corrochano

**Affiliations:** ^1^ Neurological Disorders Group, Hospital Clínico San Carlos, Instituto de Investigación Sanitaria Hospital Clínico San Carlos (IdiSSC), Madrid 28040, Spain; ^2^ Department of Physiology, Anatomy and Genetics, University of Oxford, Oxford OX1 3PT, UK; ^3^ Mammalian Genetics Unit, MRC Harwell Institute, Didcot, Oxfordshire OX11 ORD, UK; ^4^ Wellcome Centre for Integrative Neuroimaging, University of Oxford, Oxford OX3 9D, UK; ^5^ Department of Medicine, Universidad Complutense de Madrid, Madrid 28040, Spain; ^6^ Mouse Imaging Centre, The Hospital for Sick Children, Toronto, ON M57 3H7, Canada; ^7^ Mary Lyon Centre at MRC Harwell, Didcot, Oxfordshire OX11 ORD, UK; ^8^ Department of Neuromuscular Diseases, UCL Queen Square Institute of Neurology, London WC1N 3BG, UK; ^9^ MRC Prion Unit at UCL, UCL Institute of Prion Diseases, University College London, London W1W 7FF, UK

**Keywords:** Paediatric FUS-ALS, Multi-system metabolism, Neurodevelopmental disorder, RNA sequencing

## Abstract

Variants in the ubiquitously expressed DNA/RNA-binding protein FUS cause aggressive juvenile forms of amyotrophic lateral sclerosis (ALS). Most FUS mutation studies have focused on motor neuron degeneration; little is known about wider systemic or developmental effects. We studied pleiotropic phenotypes in a physiological knock-in mouse model carrying the pathogenic FUSDelta14 mutation in homozygosity. RNA sequencing of multiple organs aimed to identify pathways altered by the mutant protein in the systemic transcriptome, including metabolic tissues, given the link between ALS-frontotemporal dementia and altered metabolism. Few genes were commonly altered across all tissues, and most genes and pathways affected were generally tissue specific. Phenotypic assessment of mice revealed systemic metabolic alterations related to the pathway changes identified. Magnetic resonance imaging brain scans and histological characterisation revealed that homozygous FUSDelta14 brains were smaller than heterozygous and wild-type brains and displayed significant morphological alterations, including a thinner cortex, reduced neuronal number and increased gliosis, which correlated with early cognitive impairment and fatal seizures. These findings show that the disease aetiology of FUS variants can include both neurodevelopmental and systemic alterations.

## Teaser

**Summary:** This work reinforces the fundamental role of FUS in neurodevelopment and systemic metabolism and the notion that the *FUS* gene should be considered in clinical neurodevelopmental genetic testing.

## INTRODUCTION

The fused in sarcoma (*FUS*) gene encodes an RNA/DNA-binding protein belonging to the heterogeneous nuclear ribonucleoprotein (HNRNP) family of proteins. Variants in *FUS* perturb several biological processes, including protein and RNA homoeostasis, mRNA processing and splicing, and mRNA transport and translation (Ling et al., 2013; Lagier-Tourenne et al., 2012). *FUS* variants can cause rare juvenile, aggressive genetic forms of amyotrophic lateral sclerosis (ALS), with FUS protein localisation shifting from the nucleus to the cytoplasm, accompanied by FUS cytoplasmic aggregation in motor neurons and glia of the motor cortex and spinal cord (Vance et al., 2009). Mislocalised FUS aggregates are also observed in the frontal cortex, hippocampus and striatum in the FTD-FUS subtype of frontotemporal dementia (FTD) (MacKenzie et al., 2011), in basophilic inclusion body disease (Munoz et al., 2009) and in polyglutamine diseases such as Huntington's disease (Doi et al., 2010), highlighting a wide role of FUS in neurodegenerative disorders (Deng et al., 2014).

Up to 10% familial ALS cases are caused by variants in FUS (Kwiatkowski et al., 2009). Over 50 variants in the *FUS* gene have been identified, and the majority are in, or near, the nuclear localisation signal (NLS) domain (encoded by exon 15). Because the NLS is necessary for the import of FUS into the nucleus, mutations in this region can cause mislocalisation of FUS into the cytoplasm (Naumann et al., 2018), resulting in nuclear loss of function (Ishigaki and Sobue, 2018) and cytoplasmic toxic gain of function (Sharma et al., 2016). FUS mislocalisation is found in the FUSDelta14 variant, found in a single sporadic case causing an aggressive juvenile ALS form, with onset at 20 years of age and death 22 months later (DeJesus-Hernandez et al., 2010). This variant lies in the splice acceptor site of intron 13, resulting in skipping of exon 14 and an altered reading frame in exon 15, abolishing the NLS.

A distinct ALS disease profile is frequently seen in patients carrying *FUS* variants that can include earlier onset (juvenile ALS) and relatively fast progression (Naumann et al., 2019). *FUS* variant cases are overrepresented in paediatric ALS (Picher-Martel et al., 2020). Emerging reports now describe other phenotypes associated with *FUS* variants including learning and intellectual impairments sometimes coincidental with ALS (Yamashita et al., 2012; Lanteri et al., 2021), and such deficits can be shown by structural alterations in magnetic resonance imaging (MRI) brain scans (Hirayanagi et al., 2016). A recent categorisation of the clinical phenotypes found in *FUS*-ALS cases distinguished three clinical groups: A, axial ALS with mid-to-late disease onset; B, mild ALS with late onset and slow progression; and C, the most frequent and severe group, characterised by very-early-onset, bulbar juvenile ALS that is often preceded by learning and cognitive impairments (Grassano et al., 2022). According to this categorisation, the FUSDelta14 variant falls into the C-group clinical phenotype.

Other neurological alterations associated with FUS variants include chorea (Flies and Veldink, 2020), dementia (Goodwill et al., 2021; Van Langenhove et al., 2010), essential tremors (Merner et al., 2012) and myoclonic seizures (Dodd et al., 2019). Thus, FUS may have a critical role in the correct morphology and functioning of the brain. Recent investigations demonstrated that FUS mutation altered local mRNA translation in axons and dendrites (Blanco-Urrejola et al., 2021; López-Erauskin et al., 2018), and that FUS is needed for correct synapse function in the brain (Udagawa et al., 2015). FUS mislocalisation reportedly also caused synaptic disruptions and reduced dendrite arborisation, which altered brain connections (Sahadevan et al., 2021; Scekic-Zahirovic et al., 2021). Further studies are needed to clarify the role of FUS in the development and maintenance of brain structure and function, and in the context of neurological disease.

Despite FUS being a ubiquitously expressed protein, most studies investigating FUS-associated pathological mechanisms are conducted in neuronal cells or tissues, although a limited number of reports have investigated muscle (Picchiarelli et al., 2019; Yu et al., 2022); thus, the pleiotropic effects of FUS mutation remain largely unexplored. Because ALS and other neurodegenerative disorders are accompanied by systemic metabolic alterations, it is essential to understand the role of extraneuronal tissues in the disease. *FUS* variants have previously been associated with lipid metabolic alterations (Zhou et al., 2022; Burg et al., 2021; Rossaert et al., 2019), but the origin of such phenotypes is unclear. Therefore, further study of how *FUS* variants may perturb cell function in peripheral tissues and organs, and whether these effects directly contribute towards the aetiology of ALS/FTD, is needed (Burg et al., 2021).

Here, we aimed to further study the impact of *FUS* variants in peripheral metabolic tissues, as well as in the development and maintenance of the nervous system. To investigate this, we studied a gene-targeted mouse model carrying the FUSDelta14 mutation at the endogenous mouse *Fus* locus, together with humanisation of exon 15, which precisely recapitulates the frameshifted C-terminus observed in the variant human protein (Devoy et al., 2017). The use of knock-in models gives physiological relevance, especially with regard to understanding systemic and early-stage pathological changes, given that expression of the mutant gene is under the control of the endogenous promoter and not exogenously driven. Heterozygous FUSDelta14 (*Fus^Δ14/+^*) mice exhibit mild, late-onset neuromuscular and motor phenotypes that recapitulate aspects of ALS, but do not progress to end-stage disease within the lifespan of the mouse (Devoy et al., 2017). No metabolic or cognitive/behavioural phenotypes were observed in these heterozygous mice (Z.A., unpublished data). As a means to accelerate and reveal phenotypes associated with mutant FUS, we bred homozygous FUSDelta14 (*Fus^Δ14/Δ14^*) mice. We observed developmental structural modifications in the brain and a set of systemic changes and metabolic dysfunctions, which are correlated with transcriptional alterations in five different tissues: brain frontal cortex, lumbar spinal cord, liver, tibialis anterior (TA) muscle and brown adipose tissue. Distinct biological processes and genes were altered in different tissues, with only three genes, which are involved in DNA and RNA regulation, commonly affected. This study suggests that *FUS* mutation leads to pleiotropic phenotypes beyond the nervous system, including alterations in lipid metabolism, which are important to consider in FUS-related neurological disorders. Nevertheless, the most impacting alterations induced by FUS mislocalisation were found in the structural development of the brain, supporting the crucial role of FUS in developing and/or maintaining the correct architecture of the central nervous system.

## RESULTS

### Generation of FUSDelta14 homozygous mice and size deficit

**Fig. 1.**
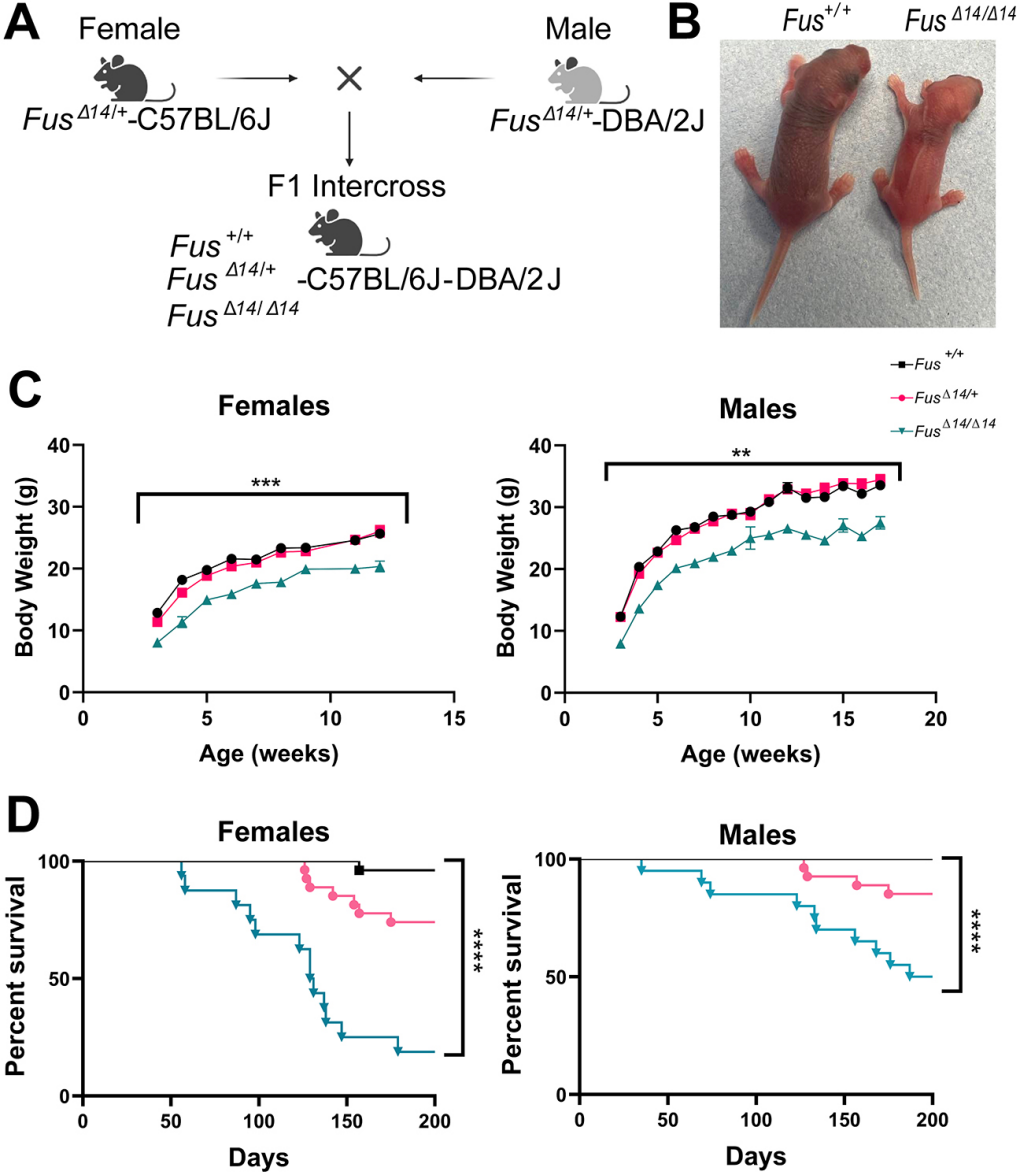
**FUSDelta14 homozygous mice are proportionately smaller than wild-type mice from development.** (A) Heterozygous mice were backcrossed for at least ten generations to place the FUSDelta14 mutation on two congenic C57BL/6J and DBA/2J backgrounds. Heterozygous mice from the two congenic lines were crossed to produce viable C57BL/6J; DBA/2J F1 homozygotes. (B) Representative pictures of postnatal day (P3) *Fus*^+/+^ and *Fus^Δ14/Δ14^* littermate mice, showing the effect of the mutation on the size of the mice. (C) Graph showing weekly body weight measurements from 6 weeks of age onwards in both sexes. Homozygous *Fus^Δ14/Δ14^* mice weighed significantly less than their heterozygous and wild-type littermates. Data are shown as the mean±s.e.m. and were analysed using two-way ANOVA followed by Tukey multiple comparisons test. Females: *n*=16 *Fus*^+/+^, *n*=17 *Fus^Δ14^*^/+^, *n*=14 *Fus^Δ14/Δ14^*. Males: *n*=14 *Fus*^+/+^, *n*=20 *Fus^Δ14/+^*, *n*=14 *Fus^Δ14/Δ14^*. (D) Kaplan–Meier survival curves of females and males show that homozygous mice start dying from seizures from 12 weeks of age. Females: *n*=26 *Fus*^+/+^, *n*=26 *Fus^Δ14/+^*, *n*=16 *Fus^Δ14/Δ14^*. Males: *n*=26 *Fus*^+/+^, *n*=27 *Fus^Δ14/+^*, *n*=16 *Fus^Δ14/Δ14^*. **P<0.01, ***P<0.001, ****P<0.0001.

Homozygous FUSDelta14 (*Fus^Δ14/Δ14^*) mice are non-viable on the C57BL/6J genetic background on which they were produced (Devoy et al., 2017) and die perinatally. To overcome this lethality, we backcrossed the line to the DBA/2J genetic background for over ten generations to determine whether changing the genetic background could overcome the lethal phenotype. Intercrossing female *Fus^Δ14/+^-*C57BL/6J mice with male *Fus^Δ14/+^-*DBA/2J mice indeed yielded viable homozygous offspring at normal Mendelian ratios (Fig. S1A). Only F1 hybrid B6-DBA animals were used in this study, and thus all animals share an identical heterozygous genetic background (Fig. 1A).

FUSDelta14 homozygotes (*Fus^Δ14/Δ14^*) were significantly smaller from birth, compared to heterozygous and wild-type littermates, indicating a developmental defect (Fig. 1B), and they remained proportionally smaller throughout their lives (both males and females) (Fig. 1C). Survival of *Fus^Δ14/Δ14^* mice was notably compromised owing to fatal seizures occurring from 12 weeks of age, showing a slightly higher frequency in females than in males (Fig. 1D). Thus, we decided to use 12 weeks of age as a humane endpoint in this study, to avoid severe adverse effects. Fatal seizures were also observed in some heterozygous mice at later time points, not only in this hybrid background (B6-DBA) but also in some heterozygous mice on the congenic C57BL/6J and DBA/2J backgrounds (J.M.G.-C., unpublished data). Importantly, seizures are also observed in other mouse models of *FUS* (Korobeynikov et al., 2022) and in FUS-ALS patients (Picher-Martel et al., 2020), inferring that *Fus* mutations, even in heterozygosity, affect brain functionality and could cause seizures.

### FUS is expressed in multiple tissues and the mutant FUSDelta14 protein is mislocalised outside the nucleus in most cell types

Because *FUS* variants cause motor neuron death and early cognitive impairments in patients, the majority of previous studies have focused on the role of FUS and *FUS* variants in the central nervous system. Given that mice are smaller from birth and that many ALS patients suffer from metabolic alterations, we decided to further investigate the role of FUS in peripheral tissues and compare it with its role in the nervous system.

First, we checked expression levels of FUS protein in peripheral tissues in adulthood, to establish in which tissues *FUS* variants might have more impact. Publicly available data (https://www.proteinatlas.org/ENSG00000089280-FUS/tissue) show that FUS is ubiquitously expressed, with peak expression levels during development (Huang et al., 2010) and predominantly nuclear localisation. To confirm these protein expression patterns and determine the effects of the mutation, we analysed FUS protein expression levels in wild-type and mutant mice at 9 weeks of age, comparing the nervous system (brain frontal cortex and spinal cord) with TA muscle and liver. We selected the frontal cortex and spinal cord neuronal tissues given their known vulnerability to *FUS* variants, TA muscle given that muscles are also prominently affected in ALS patients, and liver as a representative major metabolic organ given that pathological metabolic alterations are observed in ALS-FTD disorders.

**Fig. 2.**
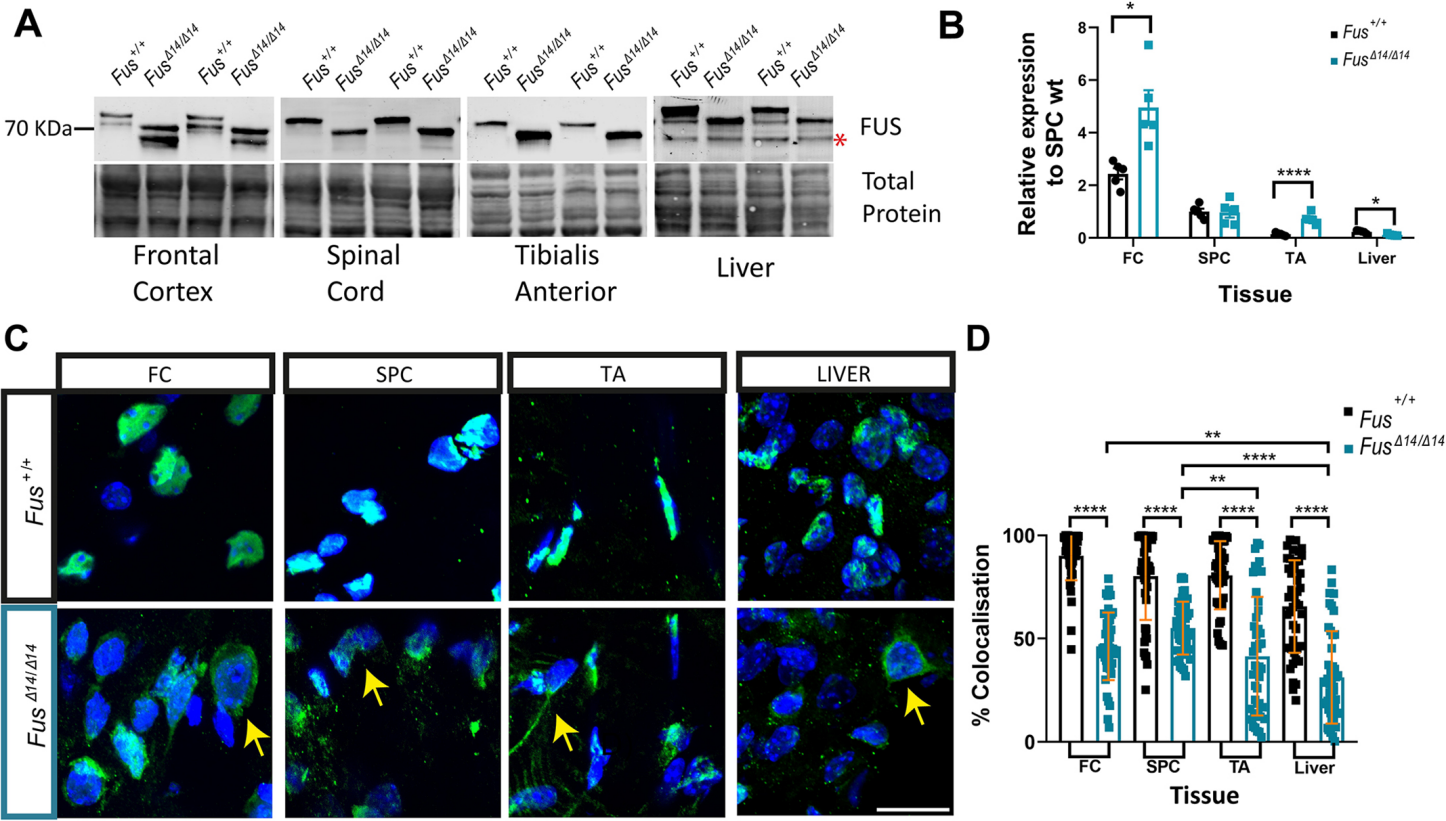
**FUSDelta14 mutation causes widespread FUS mislocalisation into the cytoplasm.** (A) Western blot analysis showing the expression of wild-type and mutant FUS protein in tissue homogenates from frontal cortex (FC), spinal cord (SPC), tibialis anterior (TA) muscle and liver (*n*=5 wild-type and mutant mice at 9 weeks of age. The asterisk marks a non-specific band in the liver. (B) Graphical representation of the semi-quantitative measures of FUS protein levels normalised by total protein and relative to those in wild-type SPC. Both bands are quantified. (C) Representative images of cellular distribution of FUS protein in various tissues at 9 weeks of age. FUS is shown in green; nuclei of the cells stained with 4′,6-diamidino-2-phenylindole (DAPI) are shown in blue. Yellow arrows point to FUS mislocalisation in the cytoplasm. Scale bar: 10 µm. (D) Graph showing the quantification of FUS mislocalisation in different tissues at 9 weeks of age as the percentage colocalization with DAPI. Individual cell measures per tissue analysed are shown (*n*=3 each genotype). Data represent the mean±s.d. Data were analysed using two-way ANOVA. *P<0.05, **P<0.01, ****P<0.0001.

Wild-type FUS protein level in adulthood varies between tissues, and we found highest expression in the frontal cortex, compared to that in the other three tissues analysed (Fig. 2A,B). Interestingly, expression of the mutant protein was significantly higher than that of the wild-type protein in the majority of tissues analysed (Fig. 2B), consistent with known autoregulatory mechanisms governing FUS expression (Humphrey et al., 2020).

We next examined cytoplasmic mislocalisation across different tissues, given that the FUSDelta14 mutation is among a group of mutations that abolish the nuclear localisation signal (NLS) domain (DeJesus-Hernandez et al., 2010; Devoy et al., 2017). In heterozygous mice, mislocalisation of FUS in the cytoplasm was observed, with FUS simultaneously remaining present in the nucleus, as expected, given the presence of a non-mutated allele (Devoy et al., 2017; Korobeynikov et al., 2022). Here, we evaluated the extent of mislocalisation in homozygotes, expressing only mutant FUS, via immunostaining in frontal cortex, spinal cord, liver and muscle from wild-type and homozygous *Fus^Δ14/Δ14^* mice (Fig. 2C). In wild-type tissues, FUS was found overwhelmingly in the nucleus of most cells (Fig. 2D). In homozygous *Fus^Δ14/Δ14^* mice, we observed notable and significant cytoplasmic mislocalisation of mutant FUS protein in all tissues, although not all cells within mutant tissues showed cytoplasmic staining (Fig. 2C,D), indicating that dysfunction caused by *FUS* variants may also have a systemic origin and not be restricted to the nervous system.

### FUSDelta14 homozygous mutation causes systemic transcriptional dysregulation

**Fig. 3.**
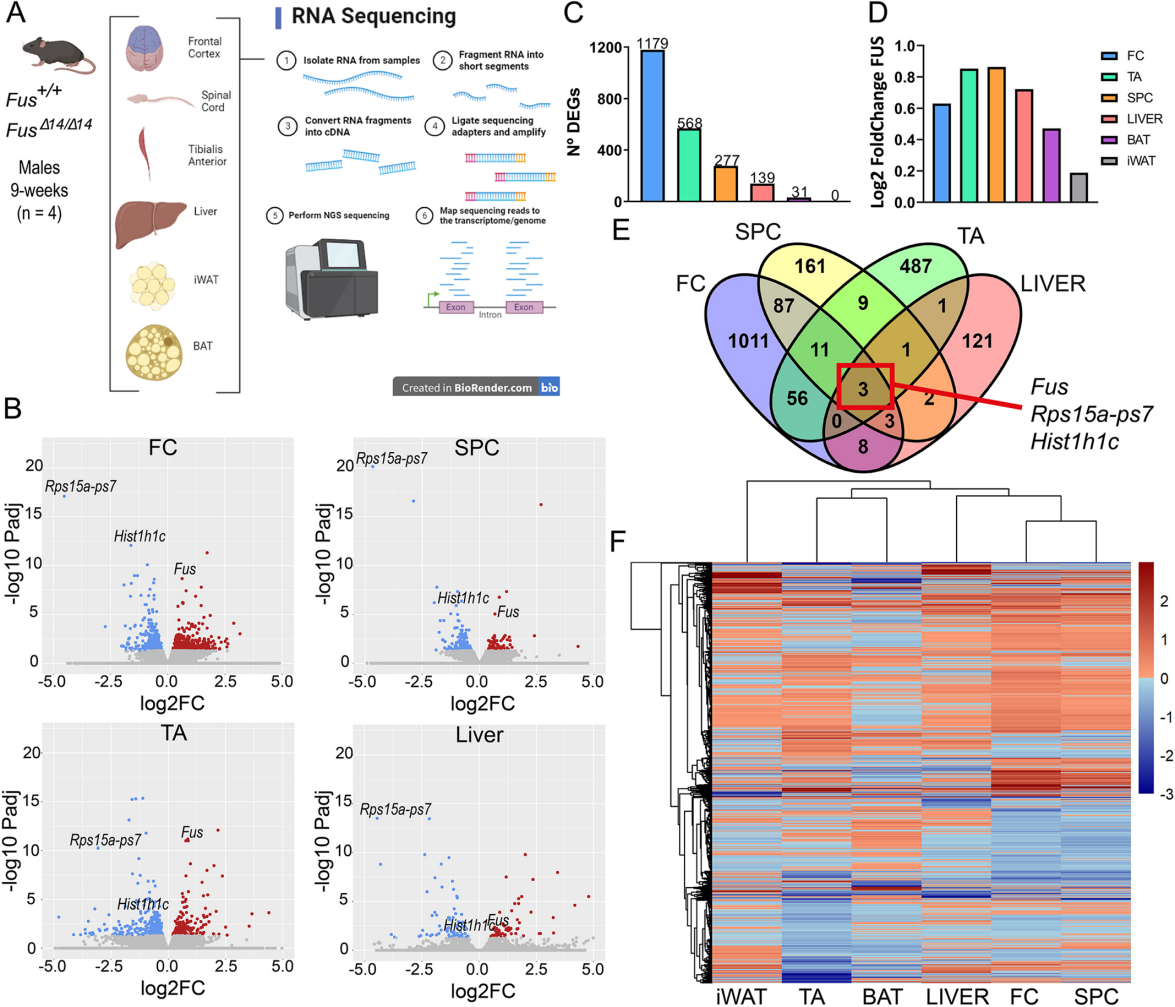
**FUSDelta14 mutation causes systemic transcriptional dysregulation.** (A) Schematic diagram illustrating the tissues used for RNA-sequencing analysis from wild-type and homozygous *Fus^Δ14/Δ14^* male mice at 9 weeks of age (*n*=4 per group). (B) Volcano plots of the four main tissues (FC, SPC, TA muscle and liver) showing the proportion of upregulated (red) and downregulated (blue) transcripts. Coloured dots denote significant differentially expressed genes (DEGs). *Fus* transcript is annotated in all plots. (C) Graph comparing the total number of DEGs (FDR<0.05) across tissues. (D) Comparison of log2 fold change values of *Fus* transcripts across different tissues in *Fus^Δ14/Δ14^* mice, versus those in wild-type mice. (E) Venn diagram representation of the common genes differentially expressed amongst four main tissues of *Fus^Δ14/Δ14^* mice, showing that only three genes are commonly dysregulated in the FC, SPC, TA muscle and liver. (F) A heatmap comparison of the genes that are upregulated or downregulated, showing that the two most similar transcriptional profiles were found in the neuronal tissues (FC and SPC) followed by the liver, brown adipose tissue (BAT) and TA muscle.

FUS protein binds to DNA to regulate gene expression in the nucleus, and also to mRNA to regulate splicing and translation. Because the FUSDelta14 mutation causes FUS mislocalisation in a majority of cells, we hypothesised that gene dysregulation may be systemic and that differential FUS expression levels may impact the degree of dysfunction in each tissue. To address this, we conducted RNA-sequencing (RNA-seq) experiments in multiple tissues, including two from the nervous systems (the brain frontal cortex and the lumbar spinal cord, primarily affected by *FUS* variants) and others that are essential for the systemic regulation of lipid metabolism [liver, muscle (TA muscle), brown adipose tissue (BAT) and inguinal white adipose tissue (iWAT)]. We studied tissues from wild-type and *Fus^Δ14/Δ14^* male mice at 9.5 weeks of age (Fig. 3A). We used only one sex (male) for the transcriptomic analysis to avoid potential heterogeneity from sexual dimorphisms, especially in the metabolic tissues. We later validated the main findings in females. In a previous transcriptomic analysis of the effect of the FUSDelta14 mutation in heterozygous mice, the spinal cord showed alterations in ribosomal and mitochondrial transcripts as early events of the neurodegenerative process (Devoy et al., 2017).

We obtained a list of differentially expressed genes (DEGs) from each tissue. DEGs at false discovery rate (FDR)<0.05 were considered statistically significant. First, most *Fus^Δ14/Δ14^* tissues showed an equal mix of upregulated and downregulated genes (Fig. 3B), except for the spinal cord and TA muscle, in which there were more downregulated genes than upregulated genes (spinal cord, 32% upregulated and 68% downregulated; TA, 39% upregulated and 61% downregulated). This is consistent with the previous transcriptomic analysis of the spinal cords of *Fus^Δ14/+^* mice, with three times more downregulated genes than upregulated genes (Devoy et al., 2017). The total number of genes affected by the FUSDelta14 mutation varied across tissues, as follows: frontal cortex, 1179 DEGs; TA muscle, 568 DEGs; spinal cord, 277 DEGs; liver, 139 DEGs; BAT, 31 DEGs (Fig. 3C; Table S1). The RNA quality and quantity from iWAT passed the quality control criteria, but the quality was inferior to that of the other tissues; therefore, there were no statistically significant DEGs with FDR<0.05, and the iWAT was removed from the main pathway analysis.

As expected, *Fus* transcript levels were upregulated in most *Fus^Δ14/Δ14^* tissues analysed, in comparison to levels in wild-type tissues, consistent with previous reports showing altered FUS autoregulation caused by *FUS* variants (Zhou et al., 2013; Humphrey et al., 2020). The level of upregulation was >0.5× wild-type levels, and was comparable (between 0.5× and 0.8×) among tissues (Fig. 3D), consistent with the higher levels of FUS mutant protein found similarly perturbed in these tissues (Fig. 2B).

Another important question was whether the FUSDelta14 mutation would affect the same target genes and pathways across tissues, which is relevant for consideration of therapies and treatments. Relatively few DEGs were discovered in BAT, and no overlapping genes were found compared to other tissues at FDR<0.05. Thus, we excluded BAT in the first comparison using DEGs at a FDR<0.05 and found only three commonly dysregulated genes [*Fus*, *Rps15a-ps7* and *Hist1h1c* (*H1f2*)] across the four tissues (Fig. 3E). We next evaluated the common DEGs among tissues using a more permissive cut-off statistical value (*P*<0.05). By lowering the threshold, we were then able to identify additional common DEGs including the BAT tissue (Fig. S2C): the same three genes as above, *Fus*, *Rps15a-ps7* and *Hist1h1c*, plus *Lag3*, *Tm4sf1*, *Pea15a*, *Xlr3a* and *Shroom4*.

As might be expected, the frontal cortex and the spinal cord shared the highest number of DEGs (104 DEGs in common). Interestingly, TA muscle shared more DEGs with the frontal cortex (70 DEGS) than with the spinal cord (24 DEGs) (Fig. S2A,B, Table S1). A heatmap analysis of gene expression showed that the frontal cortex and spinal cord exhibited the most similar transcriptional profiles, as might be expected (Fig. 3F).

### FUS mutation has pleiotropic effects on biological processes in the body

**Fig. 4.**
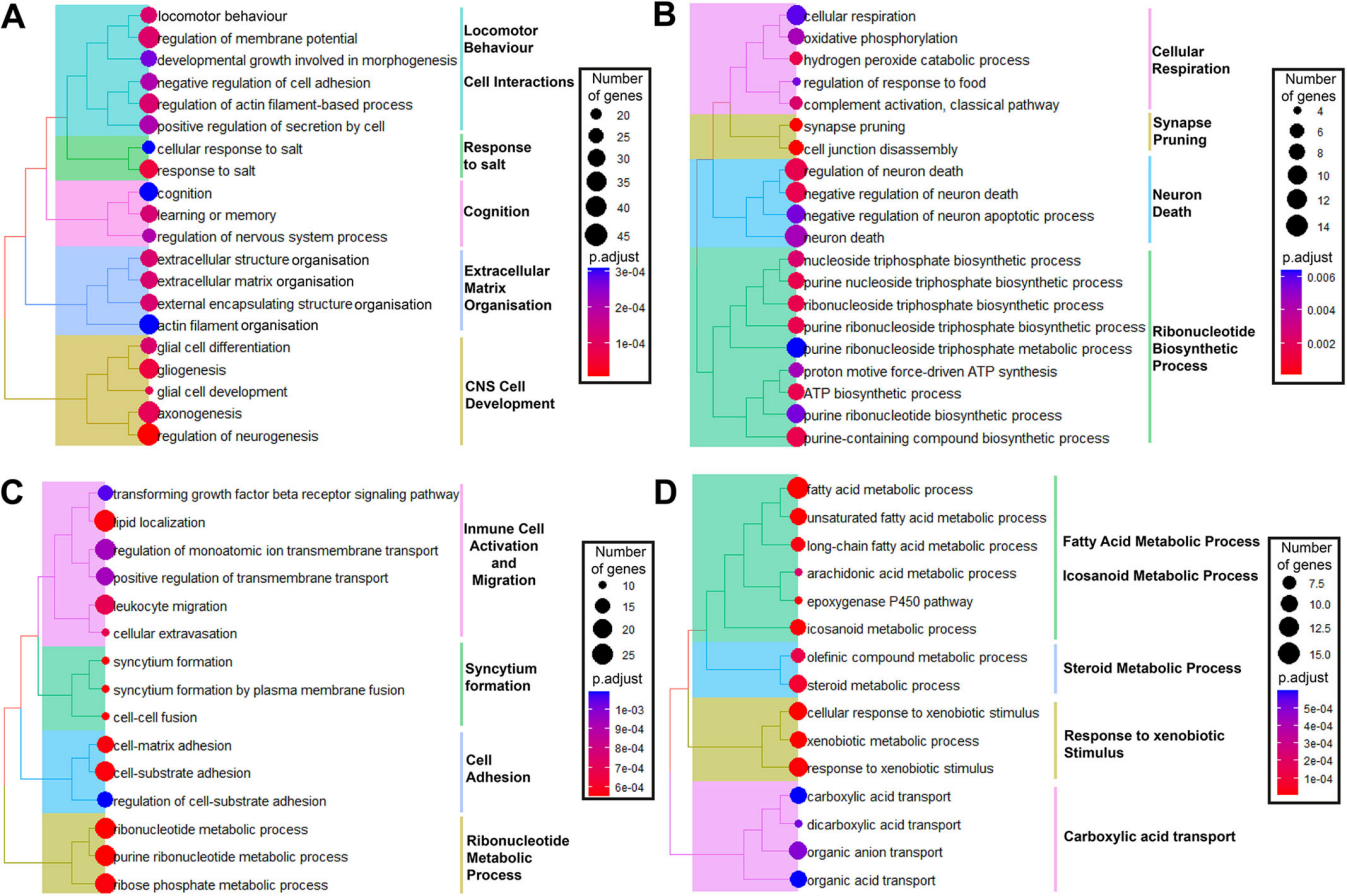
**FUSDelta14 mutation alters different biological processes in each tissue.** (A-D) Overrepresentation analysis (ORA) of the most significant deregulated biological processes based on transcriptomic data from the FC (A), TA muscle (B), SPC (C) and liver (D) of wild-type and *Fus^Δ14/Δ14^* male mice at 9 weeks of age. ORA results are represented in tree plots, with deregulated Gene Ontology (GO) terms identified, and clustered according to how many common genes are deregulated between the GO terms. The size of the dots denotes the number of genes involved in each pathways and the colour represents the level of significance (adjusted *P*-value). CNS, central nervous system.

We next investigated whether the altered biological processes could be similarly affected across tissues despite having only a few DEGs in common. Gene Ontology (GO) enrichment analysis was performed to look for biological processes associated with DEGs across the frontal cortex, spinal cord, TA muscle and liver (Fig. 4A-D). The most significant biological processes altered in the frontal cortex were related to development of the central nervous system (‘gliogenesis’, ‘axonogenesis’, ‘regulation of neurogenesis’), cognitive functions (‘cognition’, ‘learning and memory’), extracellular matrix organisation (‘extracellular structure organisation’, ‘actin filament organisation’) and structural cellular organisation (‘morphogenesis’, ‘cell adhesion’) (Fig. 4A).

The spinal cord showed distinct altered biological processes compared to the brain. In the spinal cord, the main altered processes are clustered around the production of ATP through the mitochondrial electron transport chain (‘cellular respiration’, ‘oxidative phosphorylation’, ‘ATP biosynthetic process’, ‘proton motive force-driven ATP synthesis’). The regulation of neuronal death and synapse pruning was also significantly deregulated in the spinal cord (Fig. 4B).

The main pathways and biological processes altered in TA muscle were clustered to muscle cell structure (‘syncytium formation’, ‘cell-cell fusion’, ‘cell-matrix adhesion’), ribonucleotide metabolism and immune response (‘transforming growth factor beta receptor signalling pathway’, ‘leukocyte migration’, ‘cellular extravasation’) (Fig. 4C). There were other important biological processes significantly dysregulated, such as ‘lipid localisation’, which could influence the metabolism of the muscle.

Finally, the liver showed mainly altered lipid metabolism pathways, such as fatty acid metabolism, eicosanoid metabolism and steroid metabolism processes (Fig. 4D).

We also performed gene set enrichment analysis (GSEA) to determine enriched biological processes using the full dataset of all the detected genes ranked by fold change (Subramanian et al., 2005), to identify other important pathways that might have been missed by the previous method. Only three tissues (frontal cortex, spinal cord and TA muscle) showed dysregulated pathways when using GSEA, which were mainly suppressed biological pathways (Fig. S3). The frontal cortex and spinal cord showed common inhibition of pathways related to mitochondrial ATP synthesis (Fig. S3B), which was similar to the findings from the overrepresentation analysis (ORA) in the spinal cord (Fig. 4B). The most significantly inhibited biological processes in the TA muscle were related to the immune response (Fig. S3C), which was again previously identified by the ORA results (Fig. 4C).

Taken together, these data show that the FUSDelta14 mutation affected general morphological and structural pathways (cell-cell adhesion, extracellular matrix adhesion, etc.) in most tissues, especially in the frontal cortex and TA muscle, part of which might be related to the smaller body size of the *Fus^Δ14/Δ14^* mice. There are some common pathways found in the peripheral metabolic tissues, e.g. lipid metabolic alterations in the liver and TA muscle (Fig. 4C,D). There were other pathways and processes found in neuronal and peripheral tissues, as in the case of the ribonucleotide metabolic processes, which were mainly altered in the spinal cord and TA muscle (i.e. neuromuscular) (Fig. 4B,C). Despite the few communal genes in all the tissues analysed, the FUSDelta14 mutation mainly affected specific functional pathways in each tissue.

### FUSDelta14 mutation causes alterations in brain morphology and structure

Given that FUS protein expression levels (both wild-type and mutant) were higher in the brain frontal cortex than in other tissues analysed (Fig. 2A,B), it was not surprising that the frontal cortex was also the most transcriptionally altered tissue in FUSDelta14 mutants. According to the transcriptomic data, there were several biological processes and pathways related to developmental morphogenesis, matrix organisation, neurogenesis, axonogenesis, and other cell and structural organisation pathways altered in the mutant brains (Fig. 4A). Therefore, altered brain structure and function were anticipated in homozygous mice.

**Fig. 5.**
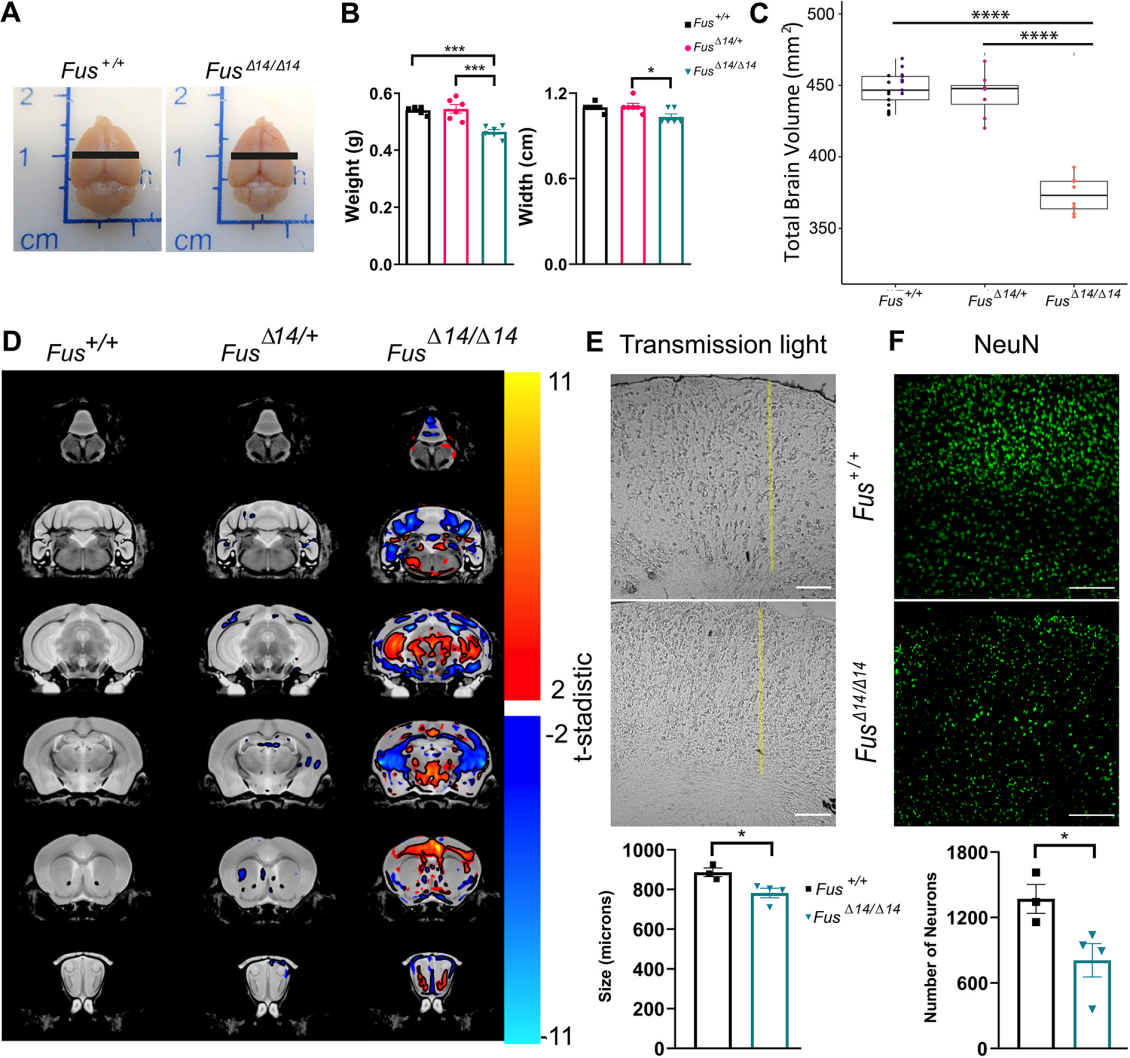
**FUSDelta14 mutation causes alterations in brain morphology and structure.** (A) Representative images of brains from female mice perfused at 12 weeks of age. Black lines show the width measurements. (B) Weight and width measurements for the three groups of mice (*n*=6 mice per group). Data were analysed using one-way ANOVA. (C) Magnetic resonance imaging (MRI) volumetric analysis of the brain in relation to terminal body weight from *Fus*^+/+^ (*n*=8) and *Fus^Δ14/+^* (*n*=8) female mice perfused at 1 year of age, and from *Fus*^+/+^ (*n*=10) and *Fus^Δ14/Δ14^* (*n*=9) female mice perfused at 12 weeks of age. Boxes represent the 25-75th percentiles, and the median is indicated. The whiskers show maximum and minimum values. (D) Representative brain sections of the relative volume changes in analysis of MRI scans from C, showing the brain regions with reduced (blue) and increased (red) relative volumes. (E) Representative light-transmitted images showing histological coronal sections of the FC of *Fus*^+/+^ (*n*=3) and *Fus^Δ14/Δ14^* (*n*=4) female mice at 10 weeks of age. Yellow lines represent the total length of the cortex. Scale bars: 100 µm. Graph shows the quantification of cortex thickness comparing the two groups. (F) Representative confocal images showing staining of histological sections with the nuclear neuronal marker NeuN (green). Scale bars: 50 µm. Graph shows the quantification of the total number of NeuN^+^ cells per area of the cortex. The data show the average number from three areas analysed per mouse, in the same areas and mice as in E. Data were analysed using unpaired two-tailed Student's *t*-test. *P<0.05, ***P<0.001, ****P<0.0001.

Brain size was smaller in *Fus^Δ14/Δ14^* mice, in proportion to the smaller body size, compared to that in wild-type littermates, for females (Fig. 5A) and males (Fig. S4A). However, *Fus^Δ14/Δ14^* brains also displayed reduced width relative to length (Fig. 5B; Fig. S4B), suggesting a structural and morphological difference in the brain. MRI analysis of *Fus^Δ14/Δ14^* female mice revealed that the total absolute brain volume was 15% smaller than that in wild-type controls (Fig. 5C). No difference in total brain volume was observed between heterozygous *Fus^Δ14/+^* mice and age-matched wild-type littermates (Fig. 5C). All brain areas of homozygous *Fus^Δ14/Δ14^* mice had reduced absolute volume compared to the same areas in control littermates. Interestingly, looking at relative volume changes (after correcting for total brain volume), much of the cortex and cerebellum was smaller in *Fus^Δ14/Δ14^* mice than in wild-type mice (Fig. 5D). This trend was not generally observed in heterozygous *Fus^Δ14/+^* mice, although there were sparse areas in the striatum and cortex regions that had relatively reduced volumes compared to those of wild-type littermates (Fig. 5D). In order to corroborate these findings, we performed histological analysis of the frontal and medial lateral cortex layers, and found that the cortex layers were significantly thinner in homozygous *Fus^Δ14/Δ14^* mice than in wild-type littermates, for females and males (Fig. 5E; Fig. S4C). The regulation of neurogenesis and gliogenesis were the top-ranked significantly altered biological processes in the frontal cortex (Fig. 4A). Accordingly, we observed a reduced total number of neurons in the frontal cortex layers (Fig. 5F; Fig. S4D). Interestingly, a previous report indicated that FUS mutation might drive the differentiation of progenitor cells towards glia instead of neurons in the brain (Stronati et al., 2021). Thus, we sought to determine whether there was an increase in astrocyte number in the mutant animals coinciding with reduced neuron number. Perfused tissues stained with the astroglial marker GFAP showed increased GFAP staining in the frontal cortex (Fig. S5A) and in the spinal cord (Fig. S5B) of *Fus^Δ14/Δ14^* mice compared to in the same regions in their wild-type controls. Those changes were associated with a mild, but significant, increase in reactive microglia, stained with the marker Iba1 (AIF1) (Fig. S5C,D), which suggested a mild reactive inflammation process in the neuronal tissues of the *Fus^Δ14/Δ14^* mice.

Taken together, these data showed that the FUSDelta14 mutation in homozygosity alters brain morphology and structure, with reduced number of neurons and increased number of glial cells, which could contribute to the incorrect functionality of the brain.

### FUSDelta14 mutation causes systemic metabolic alterations

**Fig. 6.**
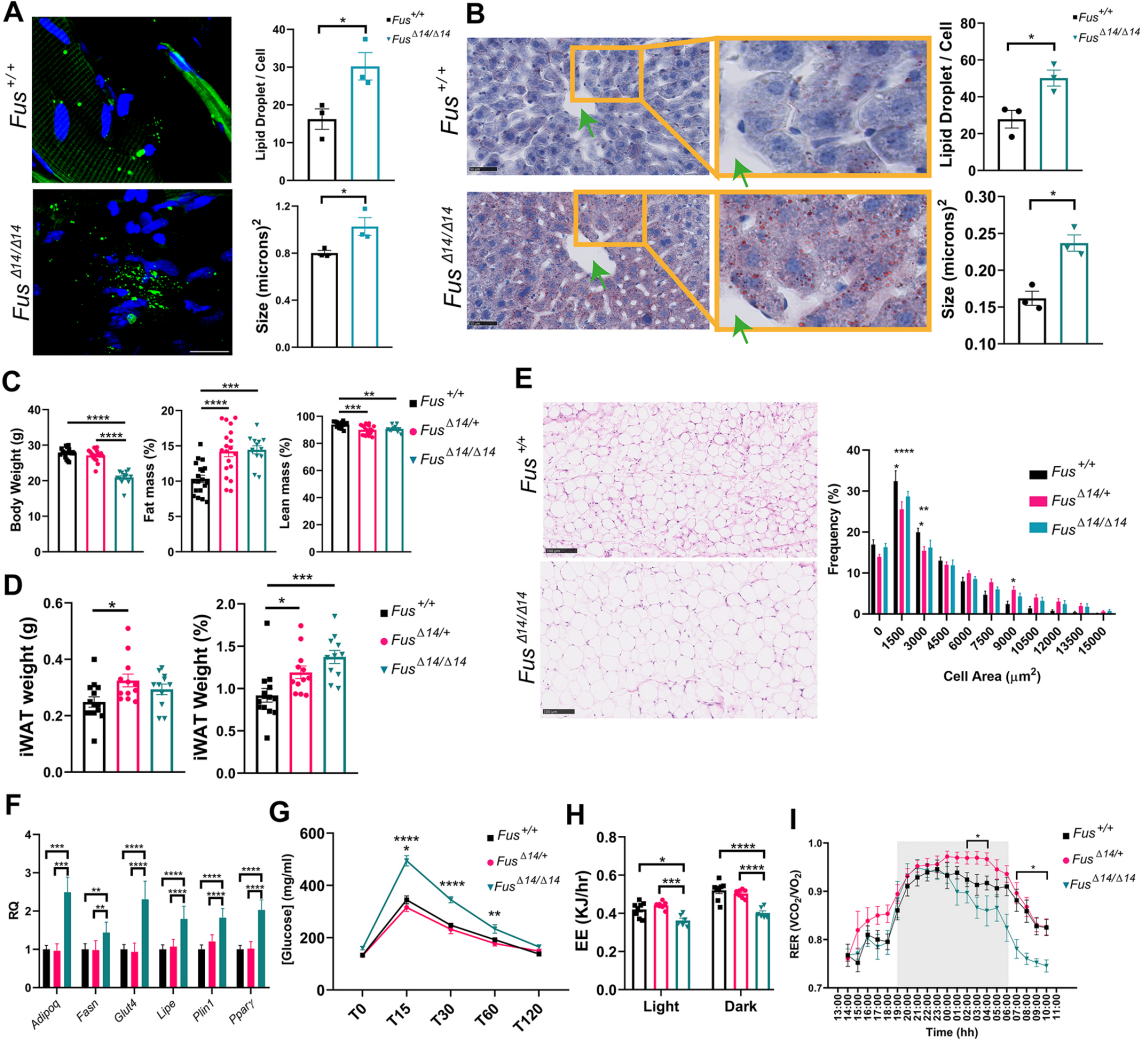
**FUSDelta14 mutation causes systemic metabolic alterations.** (A) Representative images of immunofluorescence muscle sections stained for lipid droplets (LDs) in green (with Bodipy), and quantification of LDs per cell and LD size (in µm^2^). Sections were from muscles of perfused 3-month-old female mice. Data were analysed using unpaired two-tailed Student's *t*-test. *n*=3 mice per group. Scale bar: 20 µm. (B) Representative images of histological liver sections staining for LDs in red (with Oil Red O), and quantification of LD size and LD number per area (in µm^2^). Sections from livers of perfused 3-month-old male mice. *n*=3 mice per group. Green arrows point to blood vessels. Scale bars: 50 µm. (C) EchoMRI scans of 9-week-old male mice. *n*=19 *Fus*^+/+^, *n*=19 *Fus^Δ14/+^*, *n*=13 *Fus^Δ14/Δ14^*. (D) Dissected inguinal white adipose tissue (iWAT) weights from male mice at 9 weeks. Data were analysed using the one-way ANOVA followed by Dunnett's multiple comparisons test. *n*=14 *Fus*^+/+^, *n*=12 *Fus^Δ14/+^*, *n*=11 *Fus^Δ14/Δ14^*. (E) Representative images showing iWAT histological sections with Haematoxylin–Eosin staining from females at 12 weeks of age. Scale bars: 100 µm. The histogram shows the frequency (as a percentage) of the individual adipocyte cell area (in µm^2^). Each data point represents the median value for each animal (*n*=5 mice per group), obtained from the analysis of 30 images per animal. (F) Gene expression levels of the adipogenic genes 1 week after the induction of adipogenesis in the primary pre-adipocyte culture obtained from the iWAT depots of male mice at 9 weeks of age. *n*=6 mice per group. (G) Intraperitoneal glucose tolerance test in male mice at 9 weeks of age. Data were analysed using two-way ANOVA followed by Tukey multiple comparisons test. *n*=9 per group. (H) Energy expenditure (EE) in male mice at 9 weeks of age. (I) Respiratory exchange ratio (RER) in male mice at 9 weeks of age. Data were analysed using two-way ANOVA followed by Šídák's multiple comparisons test. *n*= 8 *Fus*^+/+^, *n*= 8 *Fus^Δ14/+^*, *n*= 6 *Fus^Δ14/Δ14^*. Data in graphs represent the mean±s.e.m. *P<0.05, **P<0.01, ***P<0.0001, ****P<0.0001.

We next evaluated the consequences of the observed altered transcriptional patterns in peripheral tissues, knowing that systemic metabolic alterations are frequently observed in ALS patients (Godoy-Corchuelo et al., 2022; Zhou et al., 2022). In the liver and TA muscle transcriptome analyses, lipid metabolic processes were altered (Fig. 4C,D). In muscle histological sections, we found that there were increased lipid accumulations in female *Fus^Δ14/Δ14^* homozygous mice compared to wild-type controls (Fig. 6A). In the liver, one of the main lipid metabolic regulatory organs, the RNA-seq analysis clearly pointed towards increased lipid metabolism and lipid pathway involvement (fatty acid, eicosanoid and steroid metabolic processes). Liver histological analysis revealed increased number and size of lipid droplets (LDs) stained with Oil Red O in *Fus^Δ14/Δ14^* homozygous male mice compared to those in wild-type male mice (Fig. 6B). As expected from the liver transcriptomic profile, more LDs were observed around the blood vessels, further supporting the notion that the FUSDelta14 mutation caused systemic metabolic alterations. The liver plays an important role in the regulation of body lipid metabolism; thus, we suspected that those alterations might affect other organs and general body metabolism. This prompted us to conduct further metabolic phenotyping in these mice.

Despite homozygous *Fus^Δ14/Δ14^* mice being proportionately smaller than wild-type littermates, body composition analysis via EchoMRI revealed increased fat mass in both sexes (Fig. 6C; Fig. S6A), further evidenced by increased subcutaneous inguinal fat depots (Fig. 6D; Fig. S6B). Significantly increased fat stores were also observed in heterozygous mice. Homozygotes had only a minimal decrease in lean mass, which indicated that no substantial atrophy was present at 9 weeks of age. All these phenotypes were equally observed in homozygous males and females. To determine the cause of the increased fat stores (hypertrophy versus hyperplasia), a comprehensive histological analysis of the cell size and number was performed on perfused iWAT sections from female mice at 10 weeks of age. *Fus^Δ14/Δ14^* mice showed larger adipocytes than did *Fus^Δ14/+^* and wild-type mice, suggesting a potential hypertrophic phenotype within the adipose tissue (Fig. 6E). We then measured whether the higher accumulation of fat in the iWAT depots could also be due to inhibition of lipolysis in the *Fus^Δ14/Δ14^* mice. Male mice were challenged for lipolysis with intraperitoneal injection of the drug CL316243 (a β3-adrenergic receptor agonist) or vehicle in the control wild-type littermate group. After 1 h, we measured the levels of triglycerides and glycerol in the serum of these mice. We found no differences between wild-type and homozygous mice in their response to lipolysis challenge (Fig. S6C). We next prepared primary pre-adipocyte cultures from the iWAT depots of wild-type and *Fus^Δ14/Δ14^* male mice, and evaluated an adipogenesis maturation gene panel (Wu et al., 2021b). We found that the homozygous FUSDelta14 mutation increases adipogenesis by upregulating the expression of adipogenesis genes (Fig. 6F), thus suggesting a cell-autonomous effect of the mutation in these cells, rather than an indirect consequence of systemic alterations.

A consequence of increased subcutaneous inguinal fat stores is impaired glucose metabolism, as occurs in type 2 diabetes. We therefore evaluated whether the increased fat depots in homozygous *Fus^Δ14/Δ14^* mice could affect systemic glucose responses. We performed a glucose challenge using the intraperitoneal glucose tolerance test (IPGTT) on fasted mice. *Fus^Δ14/Δ14^* male mice had clear alterations in glucose handling, implying a slower glucose uptake rate compared to that in their wild-type and heterozygous littermates (Fig. 6G). Female *Fus^Δ14/Δ14^* mice showed a similar trend (Fig. S6D). Surprisingly, *Fus^Δ14/+^* mice showed no alteration in the IPGTT, despite showing a similar increase in the fat depots as seen in *Fus^Δ14/Δ14^* mice.

We next used indirect calorimetry to determine whether these systemic metabolic changes were linked to changes in energy expenditure. *Fus^Δ14/Δ14^* homozygous male mice displayed a lower energy expenditure rate in both the light and dark phase when corrected by lean mass (Fig. 6H), suggesting that these mice are hypometabolic. This is unsurprising considering the body composition observations (higher fat stores). These changes were not observed in *Fus^Δ14/Δ14^* homozygous female mice (data not shown). Another measurement from this *in vivo* test is the respiratory exchange ratio (RER), which is an indicator of metabolic fuel preference in tissues. A ratio of 0.7 indicates sole fat use, whereas a ratio of 1 indicates sole carbohydrate use. *Fus^Δ14/Δ14^* homozygous mice have a significantly lower RER than that of heterozygous and wild-type littermates, indicative of a shift towards lipid oxidation as a fuel preference in these mice (Fig. 6I).

These observations demonstrate major systemic lipid metabolism alterations caused by the FUSDelta14 mutation, which have a direct consequence on glucose and general metabolism. These alterations affect the correct functioning of the whole body, and also the functioning and development of the nervous system, which might partially explain the susceptibility of *Fus^Δ14/Δ14^* mice to fatal seizures observed in early adulthood.

### FUSDelta14 mutation causes cognitive, behavioural and motor alterations from early age

Finally, we looked at behavioural phenotypes and their potential association with the pleiotropic phenotypes described, with a particular focus on cognition and motor phenotypes, as these represent clinically relevant alterations observed in FUS patients. A more in-depth physiological characterisation of motor neuron function is ongoing. Thus, following observations of morphological and structural brain findings, systemic lipid metabolic alterations and transcriptional alterations in the frontal cortex, spinal cord and peripheral tissues in *Fus^Δ14/Δ14^* mice, we next assessed for behavioural deficits.

**Fig. 7.**
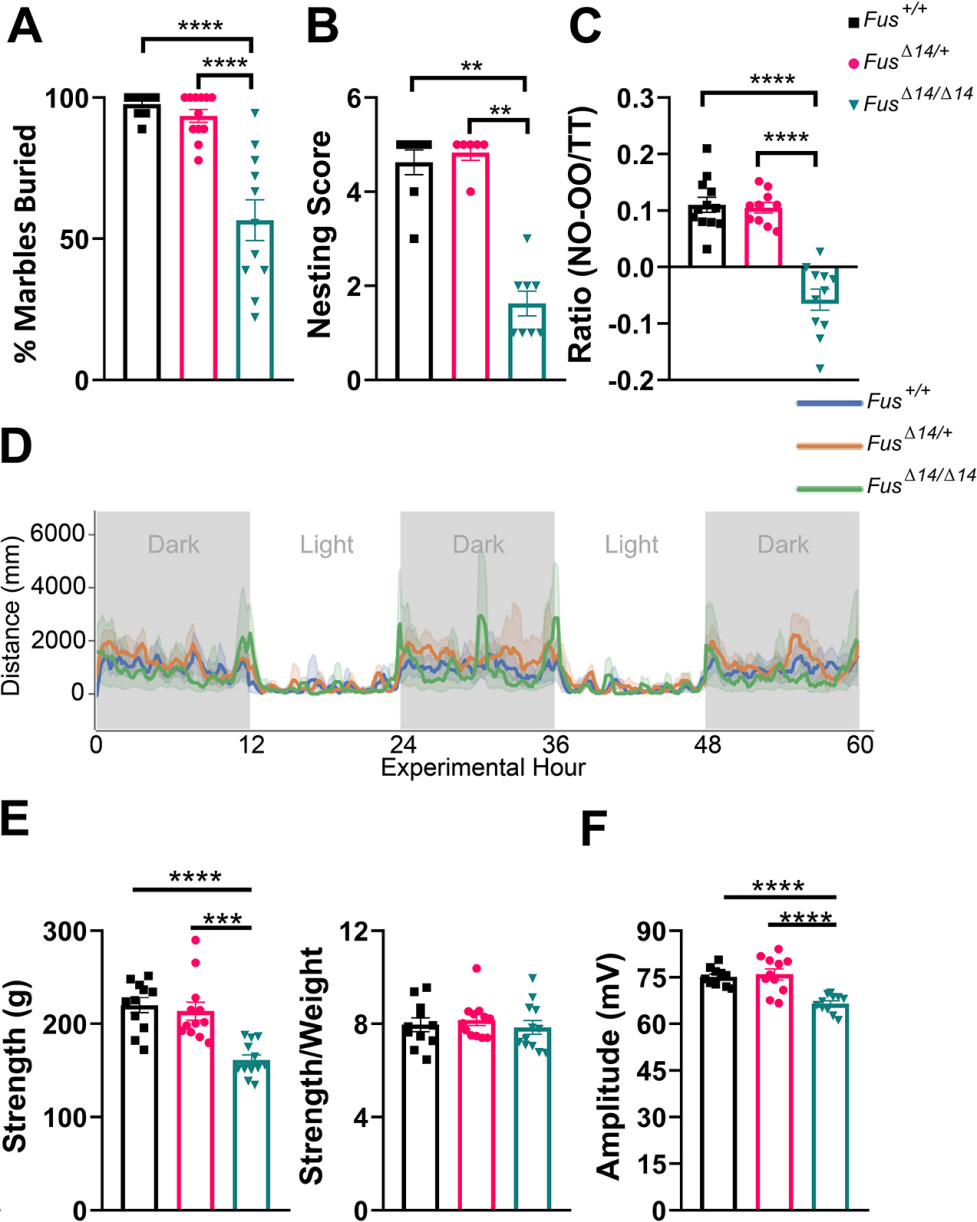
**FUSDelta14 mutation causes cognitive, behavioural and motor alterations from early age.** (A) Marble burying test. The percentages of marbles two-thirds buried were recorded. *n*=11 mice per group. (B) Nesting test. The graph shows the capacity of the mice to make a nest. *n*=8 mice per group. (C) Novel object recognition (NOR) test. To quantify the NOR result, we use a ratio that is calculated as follows: [time on the new object (NO) − time on the known object (OO)]/total test time (TT) (300 s). *n*=11 mice per group. (D) Distance moved over zeitgeber time in male-only cages of 11-week-old mice, split according to genotype, during recording session, binned into 6-min time bins and averaged over a 60-h period. The line represents mean distance over time across cages of a genotype group; the shaded error band represents 95% confidence interval. Data from individual mice within a cage were summed to produce one time series per cage. Grey-shaded areas represent darkness. Light: 13:00-19:00 and 06:00-11:00. Dark: 19:00-06:00. *n*=9 *Fus*^+/+^, *n*=10 *Fus^Δ14/+^*, *n*=6 *Fus^Δ14/Δ14^*. (E) Grip strength test. Uncorrected combined limb grip strength force and corrected means for individual animals' body weight. (F) Electromyography test. Compound muscle action potential (CMAP) amplitude in the hind limbs at 9 weeks. *n*=11 *Fus*^+/+^, *n*=12 *Fus^Δ14/+^*, *n*=12 *Fus^Δ14/Δ14^* (E,F). Data were analysed using one-way ANOVA. Data in graphs represent the mean±s.e.m. **P<0.01, ***P<0.0001, **** P<0.0001.

Owing to the susceptibility of *Fus^Δ14/Δ14^* mice to develop fatal seizures, especially noted after handling, only limited *in vivo* behavioural tests were possible for welfare considerations. Simple observational tests that measured innate mouse behaviours were preferred, such as marble burying and nesting. The exact function of the marble burying test is controversial, with the previously accepted dogma being that it measures anxiety and obsessive-compulsive disorder. Because burying, burrowing and digging are all normal behaviours in rodents, the marble burying and nesting tests are essentially a measure of mouse wellbeing (Wolmarans et al., 2016). The literature has also shown that the marble burying test could also be a measure of apathy (Keszycki et al., 2023). The novel object recognition (NOR) test uses the mouse's innate inclination to explore and is commonly used to measure aspects of learning and memory (Lueptow, 2017). *Fus^Δ14/Δ14^* mice showed innate behavioural deficits from early ages (Fig. 7A,B; Fig. S7A), performing poorly on both the marble burying and NOR tests compared to wild-type controls (Fig. 7C). From observational studies and when handling, both male and female *Fus^Δ14/Δ14^* mice also appeared hyperactive; therefore, we measured the natural activity of these mice by home cage recording and analysis. *Fus^Δ14/Δ14^* male mice had slightly increased locomotion versus that of heterozygous and wild-type littermates at 11 weeks of age (Fig. 7D). We next measured grip strength at 9 weeks of age and, similarly, found that the *Fus^Δ14/Δ14^* mice had reduced strength, but those differences disappeared when corrected by body weight (Fig. 7E). In order to discern whether there was reduced muscle force independent of body weight, we looked at the compound muscle action potential (CMAP) in hind limbs of male and female mice at 9-11 weeks of age. The electromyogram confirmed the grip strength data, with the *Fus^Δ14/Δ14^* mice having reduced CMAP compared to that of their heterozygous and wild-type littermates (Fig. 7F; Fig. S7B).

Collectively, these results demonstrate the likely developmental effect of the FUSDelta14 mutation on brain structure and morphology, neuronal and glial biogenesis, and on systemic metabolic alterations, all of which might account for the observed fatal seizures and behavioural deficits.

## DISCUSSION

Paediatric ALS is rare and not well documented. A recent review of the heterogeneous clinical symptoms reported in paediatric ALS (<18 years old) found that the majority of these cases are caused by variants in the *FUS* gene (∼52%) (Picher-Martel et al., 2020). Interestingly, learning and intellectual imapirments, and epileptic seizures were found in some of these *FUS*-ALS cases (Picher-Martel et al., 2020; Dodd et al., 2019; Yamashita et al., 2012). Further research is needed to understand the pathophysiology of these aggressive clinical forms, and the crucial role of FUS in brain development and homeostasis, as well systemic functions in the body, in order to find new potential routes for treatment of this currently incurable condition.

Here, phenotypes observed in the *Fus^Δ14/Δ14^* mouse resemble some particular paediatric FUS-ALS phenotypes, including seizures, brain structural and metabolic alterations, and cognitive and motor impairments, and serves as a biological tool for the research of aggressive paediatric disorders caused by *FUS* variants. This biological model system additionally allowed us to study the physiological role of FUS in neuronal and peripheral tissues.

Homozygosity of severe FUS-NLS mutation alleles, whereby the NLS is completely abolished, has been reported in a number of mouse models on a C57BL/6 background, and such mice typically die perinatally (or present very poor viability), and so extensive adult-stage studies were not a possibility (Korobeynikov et al., 2022; Devoy et al., 2017). Here, we were able to successfully produce viable, homozygous *Fus^Δ14/Δ14^* mice on a F1 hybrid genetic background.

The transcriptional effect of disrupting the FUS NLS has been reported from the frontal cortex and spinal cord in mice, although the reported transcriptional changes were late onset and/or limited (Devoy et al., 2017; Scekic-Zahirovic et al., 2021). Here, with the mutation in homozygosity, we identified many more significant DEGs and pathways. A previous analysis of the transcriptional alterations caused by FUS-NLS mutations at embryonic stages, in homozygosity and heterozygosity, and in mouse embryonic brain and spinal cord, pointed towards loss-of-function effects on gene expression and splicing (Humphrey et al., 2020). Because this is a homozygous mutation with a disrupted NLS, the cytoplasmic mislocalisation of the mutant FUS protein in the cells is even greater than observed in previous studies (Scekic-Zahirovic et al., 2021; Devoy et al., 2017) in which the FUS-NLS mutations presented in heterozygosity. Because the great majority of FUS-ALS patients have variants in heterozygosity, one can argue that this model might not represent a direct ALS disease model, but rather a model system that allows the study of the biological process in which FUS is involved. Here, we observed that, despite the total loss of the FUS NLS in homozygous mutant animals, nuclear FUS is still present in some cells, at varying degrees across tissues; thus, FUSDelta14 in homozygosity does not lead to total loss of function. Given the observation of abnormal FUS cytoplasmic mislocalisation across tissues, toxic loss or gain of function outside the nervous system may contribute to disease pathomechanisms. We found that although FUS is expressed at different levels across tissues, disruption to FUS autoregulation caused by the mutation is similarly affected.

One key question was whether FUS affected similar genes, transcripts or biological processes across different tissues. This is a crucial question that needs to be addressed, because most current therapies are aimed at targeting FUS in the central nervous system, without considering the systemic alterations caused by *FUS* variants. Of note, investigation of pleiotropic phenotypes in Parkinson's disease has proven illuminating, with a role for the gut-brain axis proposed as a major early pathomechanism (Wallen et al., 2022).

We found that there were few common genes and pathways affected between tissues. However, these few communal dysregulated genes could open the door to systemic FUS disease aetiology targets that require further research. One commonality was the autoregulation of *Fus* itself. The other two genes found commonly dysregulated were *Rps15a-ps7* and *Hist1h1c*. *Rps15a-ps7* is a pseudogene of the ribosomal protein S15a. In a previous RNA-seq analysis of the spinal cord of FUSDelta14 heterozygotes at 12 months of age, the gene *Rps15* was also found to be downregulated, together with many other genes related to the ribosomes (Devoy et al., 2017). The *Hist1h1c* gene encodes the histone H1.2 responsible for compaction of DNA into nucleosome structures. Coincidently, FUS is involved in DNA damage response and repair machinery (Wang et al., 2013). Interestingly, the histone H1.2 has been found translocated into the cytoplasm upon an apoptotic stimulus, such as DNA damage, where it initiates the permeabilisation of the mitochondria and apoptosis (Konishi et al., 2003). Recently, nuclear-cytoplasmic trafficking of the histone H1.2 has been found to be essential in the regulation of TBK1 and IRF3 inflammation and antiviral activity (Wu et al., 2021a). *TBK1* is another gene that causes ALS and FTD, and the TBK1 and FUS pathways might represent converging pathological pathways in disease (Gurfinkel et al., 2022). Interestingly, the *Hist1h1c* transcript was also found downregulated in the previous FUSDelta14 heterozygous analysis in the spinal cord and in the knockout of FUS in the developmental brain, supporting the role of FUS in regulating this particular gene, at different times and in different tissues. Apart from regulating the mRNA of this gene, there is evidence of FUS interacting directly with the H1.2 protein. In an interactome analysis of FUS, H1.2 had a direct protein-protein interaction with FUS (Chi et al., 2018). Further experimental work is warranted to confirm how FUS regulates the *Hist1h1c* gene and protein, and its role in the context of FUS mutations and potential contribution to disease.

Our RNA-seq data revealed the brain to be the most transcriptionally altered tissue in *Fus^Δ14/Δ14^* mice, with multiple biological processes dysregulated in relation to brain structure and function, correlating with macroscopic brain morphological changes in homozygous mice and behavioural phenotypes. Inhibitory synaptic defects and early behavioural phenotypes have also been reported in a similar mouse carrying a truncated *FUS* knock-in mutation (Scekic-Zahirovic et al., 2021) and indicated a developmental role for this gene (Zou et al., 2022). FUS is also translocated to the dendrites regulating dendrite spine morphology and synapsis, most likely by regulating the mRNA of synaptic genes localised within such structures (Blanco-Urrejola et al., 2021; Udagawa et al., 2015). Reduction in dendrites and neuronal arborisation has been reported in other mouse models of hippocampus specific FUS depletion (Fujii and Takumi, 2005; Fujii et al., 2005). It is noteworthy that we observed increased glial cells in homozygous *Fus^Δ14/Δ14^* mice compared to control littermates, with a reduced number of neurons. The transcriptional profile points towards a direct effect of FUS mutation on gliogenesis and neurogenesis. A recent study showed that the expression of another human variant of *FUS* (P525L-FUS), which is also associated with early-onset ALS, drives neuronal progenitor cells preferentially towards a glial lineage, strongly reducing the number of developing neurons (Stronati et al., 2021). Other alterations in the proliferation and differentiation of neurons have been demonstrated in FUS knockout brain and spinal cord organoids (Zou et al., 2022). Thus, with this model, we highlight the important role of FUS in development of the brain and in postnatal life (Shang and Huang, 2016), and how aggressive NLS mutations could cause cognitive alterations and seizures. Notably, some patients presenting with juvenile forms of FUS-ALS display developmental delays (Mochizuki et al., 2012; Bäumer et al., 2010; Zhou et al., 2020). Not all *FUS* variants cause developmental problems, but those that significantly target the NLS region cause the most severe phenotypes (Grassano et al., 2022). The age-related forms of frontotemporal lobar degeneration (FTLD)-FUS also display specific brain structural alterations with prominent caudate atrophy (Josephs et al., 2010), which raises the question of the role of FUS in the adult and ageing brain.

It is also remarkable that the second most transcriptionally altered tissue caused by the FUSDelta14 mutation was TA muscle. This raises the possibility that cell-autonomous changes in muscle may contribute towards pathogenesis. Motor neuron degeneration (Devoy et al., 2017) and defects in neuromuscular junctions have been seen in heterozygous *Fus^Δ14/+^* mice (Mejia Maza et al., 2021), and homozygous mice showed mild and early motor alterations. Unfortunately, we could not study the effect of ageing or long-term, progressive phenotypes in these mice owing to fatal seizures.

The ubiquitous nature of FUS expression, together with widespread transcriptional changes observed here, raises the possibility of pleiotropic phenotypes that may contribute to the overall clinical presentation in FUS-related disorders and the severe nature of FUS-ALS. This includes underlying metabolic phenotypes as observed in this study and others (Burg et al., 2021), which are consistent with metabolic changes observed in ALS/FTD patients (Godoy-Corchuelo et al., 2022). *Fus^Δ14/Δ14^* mice displayed increased fat stores, which are likely to be associated with the observed impaired glucose handling and increased lipid storage (LDs) in the liver and muscles, and a switch to fat fuel energy source preference in homozygous *Fus^Δ14/Δ14^* mice. The higher accumulation of lipids in the organs associated with impaired glucose handling seems to triggers the adaptive response of the muscles to switch to a preferred fat fuel energy source. Interestingly, these changes are partially reflected in the transcriptional profile of mutant tissues. With this approach, we could not distinguish between cell-autonomous and indirect non-autonomous effects of FUS mutation in most tissues, except in adipocytes, for which we were able to show that FUS mutation had a cell-autonomous effect, inducing adipogenesis.

It is also important to consider the impact that all these metabolic disturbances might have on the behavioural phenotypes and the occurrence of seizures found in *Fus^Δ14/Δ14^* mice (both sexes). Seizures are observed in over 200 different metabolic diseases (Rahman et al., 2013; Almannai et al., 2021), by multiple mechanisms, including neurotransmission alterations and energy deficiency and hypoglycaemia (Bartolini et al., 2023). Cognition and behavioural alterations are also known to be greatly influenced by metabolic alterations (Tanaka et al., 2020; Morys et al., 2021). Changes in body weight would probably impact the behavioural phenotypes, by affecting the general metabolic rate. Further research is needed to determine the exact mechanism linking body metabolism with the behavioural phenotypes. Similarly, muscle metabolic alterations could result in motor phenotypes (Scaricamazza et al., 2020).

Thus, *Fus^Δ14/Δ14^* mice are a biological model to study systemic alterations caused by FUS mutations and can be utilised in the search for new combination treatments considering metabolic disturbances (Jawaid et al., 2018). Here, we have demonstrated that the FUSDelta14 homozygous mutation causes FUS cytoplasmic mislocalisation in most cells and tissues of the body, with pleiotropic effects, affecting different pathways and causing systemic metabolic and neurodevelopmental phenotypes. This work reinforces that FUS-ALS may include a major neurodevelopmental component. The fundamental role of FUS in neurodevelopment supports the notion that the *FUS* gene should be included in clinical neurodevelopmental genetic testing. This study also highlights that systemic metabolic alterations should be considered as part of the disease aetiology in patients carrying *FUS* variants and further investigated for therapeutic consideration.

## MATERIALS AND METHODS

### Animals

#### Housing conditions and licensing

Mice were kept in autoventilated cages and fed *ad libitum* [Rat and Mouse Breeding 3 (RM3), Special Diet Services] with free access to water. They were maintained under constant conditions with a regular 12 h light and dark cycle, temperature of 21±2°C and humidity 55±10%, housed in same-sex cages of up to five mice. Cages contained Aspen Chips bedding (Datesand), shredded paper and a rodent tunnel for enrichment. Mice were weighed weekly and humanely sacrificed before reaching the pre-established end of 12 weeks of age. The animals were kept in their cages at all times except in the case of a behavioural test. Isolation time was kept as short as possible so as not to affect the results of other tests. Separated male and female cohorts were used in most of the analyses unless specified. The operator was unaware of sex or genotype when conducting behavioural tests.

##### At MRC Harwell

Mice were generated, maintained and studied according to UK Home Office legislation at MRC Harwell (UK Home Office Project Licence 20/0005), with local ethical approval (MRC Harwell Animal Welfare and Ethical Review Body committee) and guidelines. Assays of survival, weight, body composition, indirect calorimetry, nesting, home cage analysis (HCA) and *in vivo* lipolysis were performed at this institute.

##### In Madrid

Animals were maintained in Spain according to the guidelines of the Declaration of Helsinki. All animal procedures were approved by the Institutional Ethics Committee of Instituto de Investigación Biomédica del Hospital Clínico San Carlos, Madrid (C. I. 19/018-II, 26-11-2019) and performed in accordance with European and Spanish regulations (2010/63/EU and RD 1201/2005). Assays of survival, weight, marble burying, NOR, motor function and IPGTT were performed at this institute.

#### Mouse line generation

The FUSDelta14 mutation (g.13845A>G) at the splice acceptor site of intron 13 results in the skipping of exon 14 and an out-of-frame translation of a humanised exon 15 (FUS p.G466VfsX14), as described in Devoy et al. (2017). Heterozygous mice on a congenic C57BL/6J background, carrying the FUSDelta14 allele, were backcrossed for at least ten generations to additionally place the FUSDelta14 mutation on the DBA/2J genetic background. Subsequently, C57BL/6J heterozygous females were bred to DBA/2J males to produce viable C57BL/6J; DBA/2J F1 homozygotes (Fig. 1A) and control littermates. Only F1 hybrid animals were used in this study.

At weaning, all mice were genotyped by PCR from an ear biopsy. DNA was extracted using the Phire Tissue Direct PCR Master Mix kit (Thermo Fisher Scientific), following the manufacturer's recommendations. A 1:10 dilution of the total DNA was used to perform the PCR, with the Master Mix and appropriate primers (see list of primers in Table S2). PCR products were run on a 2% agarose gel in Tris-Borate-EDTA (TBE; Thermo Fisher Scientific, Spain) and the DNA-staining Midori Green Advance (Nippon Genetics Europe), and visualised with a gel scanner D-Digit (LI-COR Biosciences).

### Phenotyping tests

All tests were conducted in both sexes, unless otherwise stated.

#### Weight

Non-anaesthetised mice were weighed inside a beaker weekly and before appropriate behavioural tests. To correct for animal movements, an average weight was taken after 5 s.

#### Body composition

Body composition was measured by placing non-anaesthetised mice in a transparent plastic tube, which was then inserted into an EchoMRI TM-100H machine, which uses quantitative magnetic resonance technology to calculate body composition. The proportion of fat and lean mass was obtained in relation to the total body weight.

#### Indirect calorimetry

Individual animals were placed in metabolic cages connected to the PhenoMaster indirect calorimetry system (TSE Systems) for ∼20 h, including dark and light phases of the day. The system was connected to a Siemens High-Speed Sensor Unit containing oxygen and carbon dioxide sensors. The amounts of oxygen consumed and carbon dioxide released by the body were measured to determine energy expenditure and the RER. Energy expenditure values were corrected by the average genotype lean mass using two-way ANCOVA.

#### Marble burying test

Nine glass marbles were evenly spaced in rows of three in an individually ventilated cage containing ∼4 cm depth of aspen bedding. The mouse was placed in the cage, and the individually ventilated cage lid was positioned on top for 30 min. The number of marbles more than two-thirds buried was recorded.

#### Nesting test

Mice were placed in individual cages with wood-chip bedding but no environmental enrichment items. Nestlets (3 g weight) were placed in the cage overnight. Nests were assessed the next day on a rating scale of 1-5 as follows: 1, nestlet not noticeably touched (90% intact); 2, nestlet partially torn (50-90% remaining intact); 3, nestlet mostly shredded but no identifiable nest site; 4, an identifiable but flat nest (more than 90% of the nestle torn); 5, a near-perfect nest with walls higher than mouse body height.

#### NOR test

Initially, the mouse was habituated within the area of realisation (methacrylate box of 40×40 cm). The habituation process spanned 2 days, and the mouse spent 10 min in the box each day. On the third day, the test was carried out by measuring the time spent with each object. To do this, the mouse was placed in the box for 10 min with the two initial objects (two equal weights). One of the objects was removed and replaced with a new object for another 5 min. After 15 min, the mouse was removed and placed back into its home cage. The box was cleaned before each run with 70% alcohol. To quantify the NOR result, the following ratio was used: (time spent with the new object − time spent with the known object)/total time (300 s).

#### Grip strength test

Mice were placed on the grip meter (Bioseb) with all four paws on the grid. Once on the apparatus, traction was applied via the tail. The resistance the mouse applied on the grid was recorded. Each mouse had three runs. The mean value was corrected for the individual animal's body weight.

#### Muscle CMAP measurements

The protocol of Pollari et al. (2018) was followed for electromyography measurements of muscle. An analogue stimulator (model 588, Grass Telefactor) and a stimulus isolation unit (model PSIU6, Grass Instrument Co.), plus an AC/DC amplifier model 3000 (A-M Systems) were used. The recording interface was CED1401, and the software used for recording and subsequent analysis was Spike v8.04 (Cambridge Electronic Design).

Briefly, the mouse was anaesthetised and kept with isoflurane until the procedure was completed. Five electrodes were placed on the mouse positioning detail as in Pollari et al. (2018). The protocol to register the maximal responses followed increasing intensities of stimulation, starting at 1 mA up to 14 mA. After the procedure, the mouse was left in a warm cage and monitored until it fully recovered, before returning it to its cage. During the following days, the mice were monitored to check health status.

#### HCA

Radio frequency identification microchips were injected subcutaneously into the lower-left or -right quadrant of the abdomen of each mouse (separate cohort) at 9 weeks of age. These microchips were contained in standard ISO-biocompatible glass capsules (11.5×2 mm; PeddyMark). The procedure was performed on sedated mice (Isoflo; Abbott) after topical application of local anaesthetic cream on the injection site prior to the procedure (EMLA Cream 5%; AstraZeneca). The animals were allowed to recover from the microchip procedure for at least 1 week before being placed in the HCA rigs for data collection. The procedure has been described previously (Bains et al., 2016). A mounted low-profile base plate that contains a 2D array of 18 RFID antennae (in a 3×6 array) directly beneath the home cage monitored spatial location and detected activity. The software package ActualHCA-Capture (Actual Analytics) was used to capture readings from the baseplate antennae as well as synchronised video for subsequent validation work. The timeframe of interest was defined as 30 min directly preceding lights being turned on (06:30 to 07:00) and 30 min directly preceding lights being turned off (18:30 to 19:00). When analysing activity during the time frames of interest, data were summed per time bin (6 min) per mouse, and an average activity across the five time bins was calculated. Pairwise post hoc comparison tests were conducted by computing the estimated marginal means (least-squares means) for factor combinations and correcting for multiple comparisons using the Benjamini–Hochberg method to decrease the FDR. This process was run using R's ‘emmeans’ function, which returned adjusted *P*-values. These values were used to indicate the statistical significance of the genotype effect at various levels of factor combination. The full protocol can be found in Bains et al. (2016).

#### IPGTT

Mice were fasted for 12 h overnight. The mice were weighed to determine the dose of glucose to be injected. A local anaesthetic cream (Lidocaine/Prilocaine, Emla 25 mg/g of lidocaine+25 mg/g of prilocaine cream, Aspen Pharmacare) was applied to the base of the tail 30 min before the test. A small incision was made at the lateral vein of the tail to produce a drop of blood, enough to measure baseline glucose levels (0 min) with a glucose meter (ACCU-CHECK Performa meter). An intraperitoneal injection of 20 mg/kg glucose was administered to the mouse. Additional glucose measurements (mg/ml) were taken at 15, 30, 60 and 120 min.

#### *In vivo* lipolysis measurement

Mice were injected with 0.1 mg/kg CL316243 (Cayman Chemical), a β3-adrenergic receptor agonist, or phosphate-buffered saline (PBS) as a vehicle control. Blood was collected 1 h post-injection via a terminal retro-orbital collection. Blood was centrifuged at 1500 ***g*** for 10 min to isolate plasma. To measure lipolysis, free fatty acids and glycerol levels were quantified using enzyme colorimetric assays on a Beckman Coulter AU680 clinical chemistry analyser.

### Histological sections and immunostaining

Mice were terminally anesthetised with 0.4 mg/kg fentanest and 40 mg/kg thiopental, and cardiac perfused with the fixative 4% paraformaldehyde (PFA) without methanol in 1× PBS, pH 7.4. The dissected organs were post-fixed in the same fixative overnight at 4°C, and kept in 1× PBS with 0.1% sodium azide until use.

#### Histological analysis of fat depot

Perfused iWAT tissues were paraffin embedded, cut at 5 µm and stained with Haematoxilin–Eosin. The sections were then scanned, and the images were stored using NPD.view2 software. Images were taken at 20× magnification to quantify the size and number of adipocytes. Five representative images of the tissue were captured for further analysis. Adipocount software (Zhi et al., 2018) was used to obtain the total number of adipocytes along with their size. Once obtained, the data were curated, because some values might have no biological meaning. So, we excluded adipocytes less than 100 µm in diameter and those greater than 20,000 µm in diameter.

#### LD staining

##### Oil Red O staining

Livers from perfused mice were and cryoprotected with 30% sucrose until the tissue sank (∼2 days). Tissues were dried and embedded in optimal cutting temperature (OCT) compound (Thermo Fisher Scientific, Spain) using isopentane and dry ice. Using a cryostat (Leica), livers were sectioned at 12 µm, coverslipped and stored at −20°C until needed. Before staining, slides were thawed at room temperature for ∼30 min. Then, 1.5 ml of 60% isopropanol was added to each section, and sections were incubated for 5 min at room temperature. Isopropanol was then removed and ∼200 µl Oil Red O (Table S3) was added to the slides for 15 min at room temperature. The stain was then removed, and slides were washed with deionised water until clear. Sections were counterstained with Haematoxylin and coverslip mounted using an aqueous mountant. Samples were then scanned using a Nanozommer slide scanner (Hamamatsu) at 40× zoom.

##### Bodipy staining

Perfused TA muscles cryoprotected in OCT were sectioned into 80 µm sections using a cryostat (Leica) and placed into individual wells containing phosphate-buffered saline (PBS). Free-floating sections were stained using the fluorescent lipid probe Bodipy (Thermo Fisher Scientific, Spain) at a concentration of 1:600, and nuclear staining was conducted using Hoestch at a concentration of 1:1000, for 2 h at room temperature. After washes with PBS with 0.02% Triton X-100, the tissues were mounted on slides with Medi Fluorsave (Calbiochem), and images were taken on a Olympus Fluoview FV1000 confocal microscope with system version 3.1.1.9.

#### Immunohistochemistry

Tissues were cryopreserved by immersion in 30% sucrose. Tissues were dried and embedded in OCT using isopentane and dry ice, and frozen at −20°C until being cut with a cryostat (Leica). Transverse sections at 40 µm were collected through the lumbar spinal cord, brain frontal cortex, liver and muscle (TA). Free-floating sections were blocked with goat serum and incubated with primary antibody in PBS with 0.02% Triton X-100 (for the list of antibodies used, see Table S3), followed by secondary antibody (Alexa Fluor dye conjugated, Thermo Fisher Scientific), and then mounted together with Hoestch stain to detect nuclei. Images were taken using a Olympus Fluoview FV1000 confocal microscope with system version 3.1.1.9.

Consecutive area-specific images were acquired in the frontal cortex of the brain in the bregma region and in the ventral horn of the spinal cord. The images were then loaded into Fiji/ImageJ for intensity quantification and colocalisation analysis. The ‘Colocalisation Threshold’ macro in the Fiji software was used to calculate the percentage of colocalisation. For this, the images to be analysed were selected in red and green colours, the macro was selected, and the default options were used. The TM1 and TM2 values refer to how much of one protein colocalised with the other and vice versa.

### Western blot analysis

Tissues were homogenised using mechanical disaggregation with beads (Precells) in RIPA buffer (Thermo Fisher Scientfic) at 4°C with protease inhibitor cocktail (Roche). After 20 min of centrifugation at 13,000 ***g*** at 4°C, the supernatant was collected, and the total amount of protein was quantified using the DC Assay method (Bio-Rad). For each of the samples, 20 µg protein was loaded in a 10% acrylamide precast gel (Genescript) under reducing conditions, run and transferred into a PVDF low fluorescence membrane (GE Healthcare). After blocking, the membranes were incubated with anti-FUS primary antibody (1:1000; Table S3), and labelled secondary antibody (1:10,000) was added for infrared detection before scanning in the infrared scanner Clx (LI-COR Biosciences). A total protein detection kit (LI-COR Biosciences) was used following the manufacturer's instructions for the loading correction. The intensity of the bands was quantified using the image software Image Studio v.5.2 (LI-COR Biosciences).

### Structural MRI

Mice were intracardially perfused with a first flush of PBS-Gd (Gd: 2 mM Gadovist, Gadolinium contrast agent, Bayer), and then with 4% PFA-Gd. The brains were obtained with the skull intact and kept for 24 h in 4% PFA-Gd at 4°C and stored until use in a solution made of PBS with Gd and sodium azide at 4°C. All mice studied with MRI were females. Samples from nine *Fus^Δ14/Δ14^* mice of 10-12 weeks of age and nine age-matched wild-type littermates were scanned in a multi-channel 7.0 Tesla MRI scanner (Agilent Inc.) using a custom-built 16-coil solenoid array (Bock et al., 2005). A T2-weighted 3D fast spin-echo (FSE) sequence with cylindrical k-space acquisition sequence was used (Spencer Noakes et al., 2017), with the following parameters: repetition time (TR), 350 ms; echo time (TE), 12 ms; echo train length (ETL), 6; effective TE, 30 ms; two averages; field of view (FOV)/matrix size, 20×20×25 mm/504×504×630; total imaging time, 14 h. Samples from eight *Fus^Δ14/+^* mice and eight age-matched wild-type littermates, at 1 year of age, were scanned in a 7-Tesla 306 mm horizontal bore magnet (BioSpec 70/30 USR, Bruker). Eight samples were imaged in parallel using a custom-built 8-coil solenoid array and the same scan parameters as for *Fus^Δ14/Δ14^* samples, but with four effective averages and FOV/matrix size of 20.2×20.2×25.2 mm/504×504×630, with total imaging time of 13.2 h. For all samples, the resulting images had isotropic resolution of 40 µm. All images were registered together using pydpiper (Nieman et al., 2018). Voxel volumes were estimated from the Jacobian determinants and modelled as a function of genotype and cohort. Absolute volume changes in mm^3^ and relative volume changes, measured as percentages of the total brain volume, were compared. Differences were considered significant for *P<*0.05 after FDR correction.

### Quantitative PCR analysis

Fresh dissected tissues were snap frozen and stored at −80°C until required. Tissues were taken at 9 weeks of age from male wild-type and homozygous *Fus^Δ14/Δ14^* mice. RNA was extracted from tissues using an RNeasy Lipid Tissue Mini Kit (Qiagen). Using the extracted RNA, cDNA synthesis was performed using a High Capacity cDNA Reverse Transcriptase Kit (Thermo Fisher Scientific) with 2 µg total RNA. cDNA for quantitative PCR reactions was used at a final concentration of 20 ng per well. Fast SYBR Green Master Mix (Thermo Fisher Scientific) was added, followed by the appropriate primer, with a final well volume of 20 µl. Primers are listed in Table S2. All reactions were run in triplicate.

### RNA-seq

RNA was extracted from frontal brain, lumbar spinal cord, BAT, iWAT, TA muscle and liver of wild-type and homozygous *Fus^Δ14/Δ14^* male mice at 9 weeks of age. Quality and quantity was assessed using the RNA Nano 6000 Assay Kit of the Bioanalyzer 2100 system (Agilent Technologies). Sequencing libraries were generated using an NEBNext^®^ UltraTM RNA Library Prep Kit for Illumina^®^ (NEB) following the manufacturer's recommendations, and index codes were added to attribute sequences to each sample. The clustering of the index-coded samples was performed on a cBot Cluster Generation System using PE Cluster Kit cBot-HS (Illumina) according to the manufacturer's instructions. After cluster generation, the library preparations were sequenced on an Illumina platform, and paired-end reads were generated. The total number of reads was 30 million per sample. Library preparation and RNA-seq was carried out by Novogene.

### Bioinformatics analysis

Reference genome and gene model annotation files were downloaded from a genome website browser (NCBI/UCSC/Ensembl) directly. Indexes of the reference genome were built using STAR, and paired-end clean reads were aligned to the reference genome using STAR (v2.5). HTSeq v0.6.1 was used to count the read mapped to each gene. For normalisation, the fragments per kilobase per million mapped fragments (FPKM) of each gene were then calculated. Differential expression analysis between genotypes was performed using the DESeq2 R package (2_1.6.3). The resulting *P*-values were adjusted using Benjamini and Hochberg's approach for controlling the FDR. Genes with an adjusted *P*-value <0.05 found by DESeq2 were assigned as differentially expressed.

To identify which biological processes were altered from the RNA-seq study, two different techniques were used: ORA and GSEA. ORA is a statistical method that determines whether genes from pre-defined sets, those belonging to a specific GO term, are present more than would be expected (overrepresented) in a subset of data. The ORA was performed using the clusterProfiler package (v3.16.1) in R. As input it receives all (both upregulated and downregulated) DEGs from DESeq2, obtained using the cut-off criteria for statistical significance adjusted *P-*value <0.05.

GSEA is a genome-wide expression profile chip data analysis method for identifying functional enrichment through a comparison of genes and predefined gene sets (Subramanian et al., 2005). The GSEA was performed using the clusterProfiler package (v3.16.1) in R. As input it receives all ranked genes by fold change from DESeq2 analysis. The significance of enriched gene sets was estimated by FDR q-value.

### Statistical analysis

Statistical analysis was conducted using GraphPad Prism and SPSS. Two groups were compared at a single time point using unpaired two-tailed Student's *t*-test. Body weights were compared between genotypes, across multiple time points, using two-way ANOVA repeated measures/mixed models and Bonferroni correction of multiple testing. Two groups were compared across multiple time points using two-way ANOVA with Šídák's multiple comparisons post hoc test. Three or more groups were compared at a single time point using one-way ANOVA with Dunnett's post hoc test. Statistical analysis of quantitative reverse transcription PCR data was performed on ΔCT values. Two-way ANCOVA (SPSS) was used to correct the energy expenditure data for the lean mass interaction. Statistical significance was defined as *P*<0.05 for analysis of phenotyping and molecular biology data, and adjusted *P*<0.05 for analysis of differentially expressed transcripts in RNA-seq data (statistical values for the latter generated with DEseq2). Please see figure legends for sample size *n* numbers (*n* numbers refer to biological samples, i.e. numbers of animals used in animal experiments). Statistical details for each experiment can be found in the figure legends. For adipocyte size statistics, two-way ANOVA and Tukey's correction for multiple groups were used. Statistical analysis was performed and figures of MRI data were prepared in RStudio using RMINC, MRIcrotome, data tree, tidyverse, ggplot2, grid, dplyr, viridis, plotrix and graphics packages.

All graphs were generated using GraphPad Prism 9. Venn diagrams were created using Venny2.1. BioRender.com was used to create original diagrams/figures.

## Supplementary Material

10.1242/dmm.050200_sup1 Supplementary information

Table S1. List of DEGs in all tissues.
