# Supplementary material for: Mutation in the FUS nuclear localisation signal domain causes neurodevelopmental and systemic metabolic alterations
Source: Dis Model Mech. 2023 Oct 23;16(10):dmm050200. doi: 10.1242/dmm.050200 (PMC10642611; doi:10.1242/dmm.050200)
Supplement: Supplementary information [file dmm-16-050200-s1.pdf]

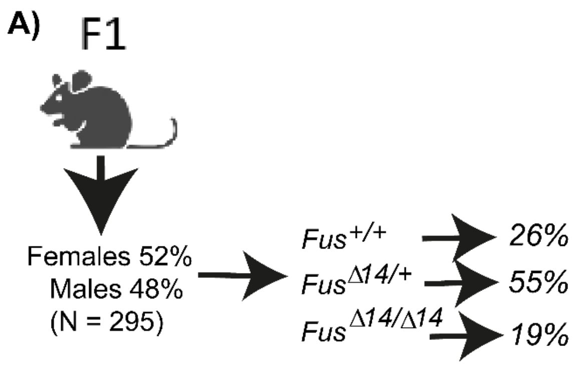

Fig. S1. Breeding F1 intercrossed ratios. Body weight and survival of Males. A. Ratios of males and females born out of the 295 mice born from the F1 intercrossed breeding, showing 52% female and 48% male. The three genotypes are produced in normal ratios.

Disease Models & Mechanisms • Supplementary information

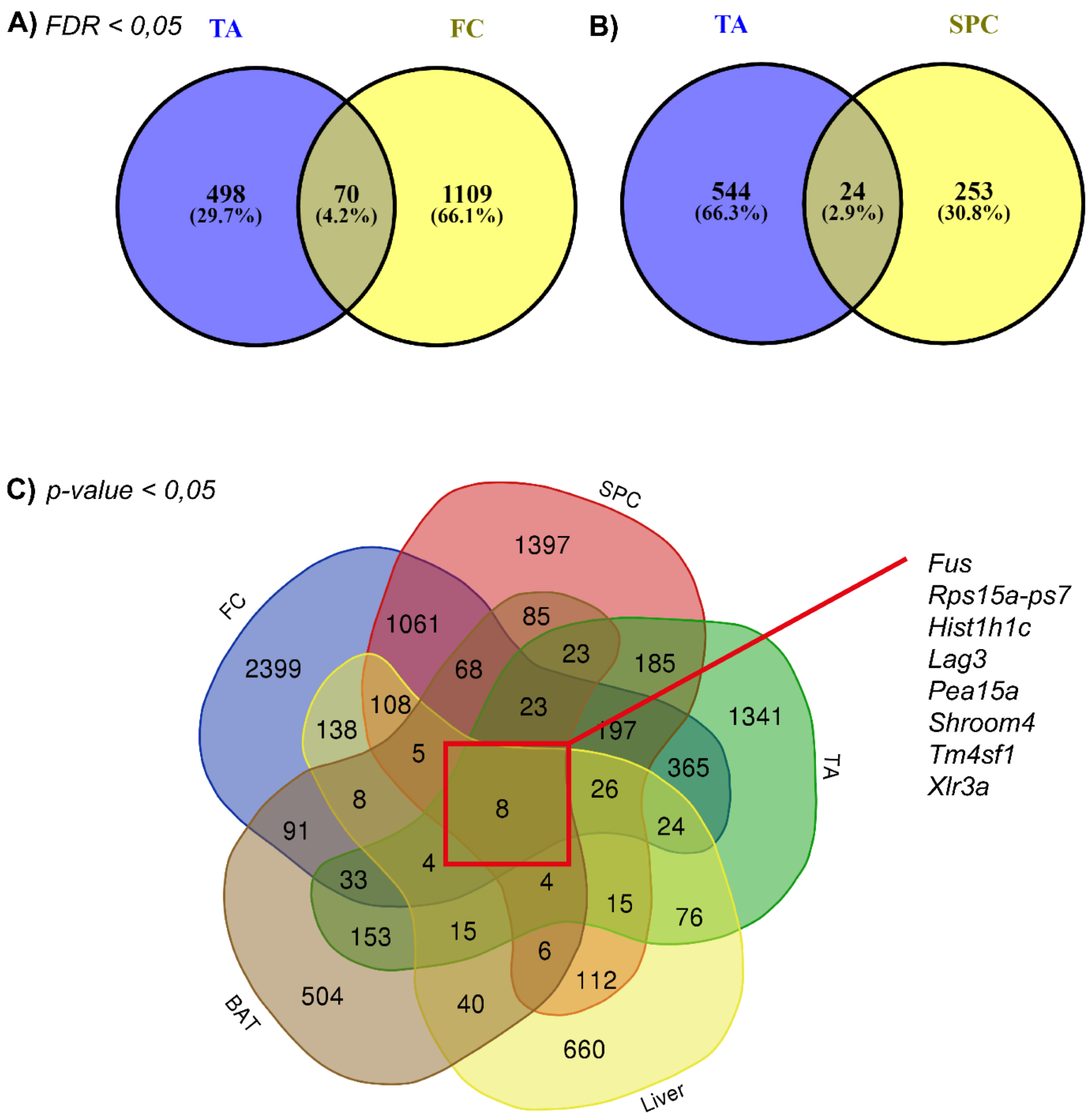

Fig. S2. Venn diagrams showing common DEGs between tissues. A. Venn diagrams showing the common DEGs between the TA and the frontal cortex. **B.** Venn diagrams showing the common DEGs between the TA and the spinal cord. **C.** Venn diagram showing DEGs in common between the 5 tissues (frontal cortex, spinal cord, BAT, TA muscle and liver), using p-value< 0.05. The 8 commonly dysregulated genes are shown in the red box.

Disease Models & Mechanisms • Supplementary information

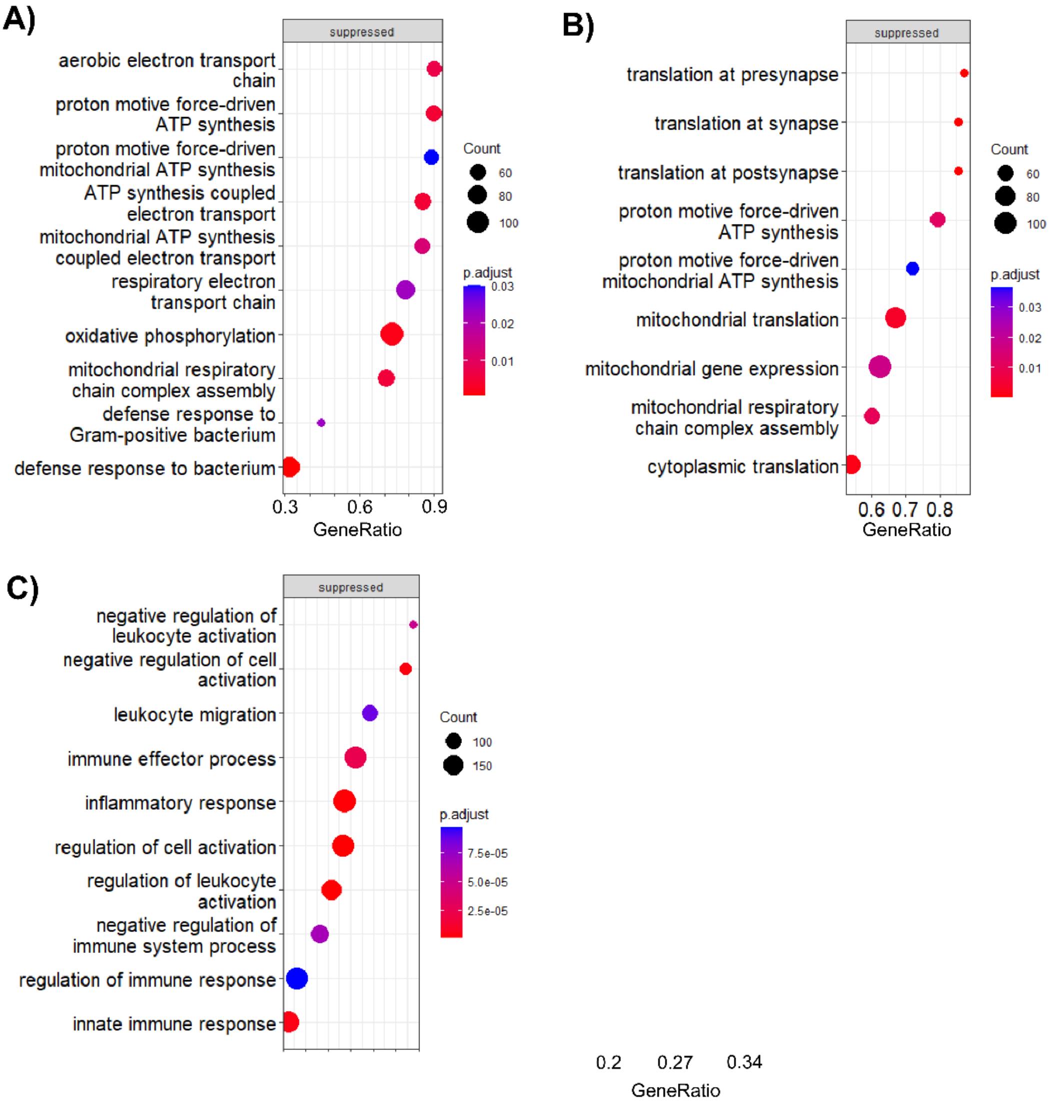

Fig. S3. GSEA from three tissues representing the most significant biological pathways and processes using Dot plots. A. Frontal cortex. **B**. Spinal cord, **C.** Tibialis anterior muscle.

Disease Models & Mechanisms • Supplementary information

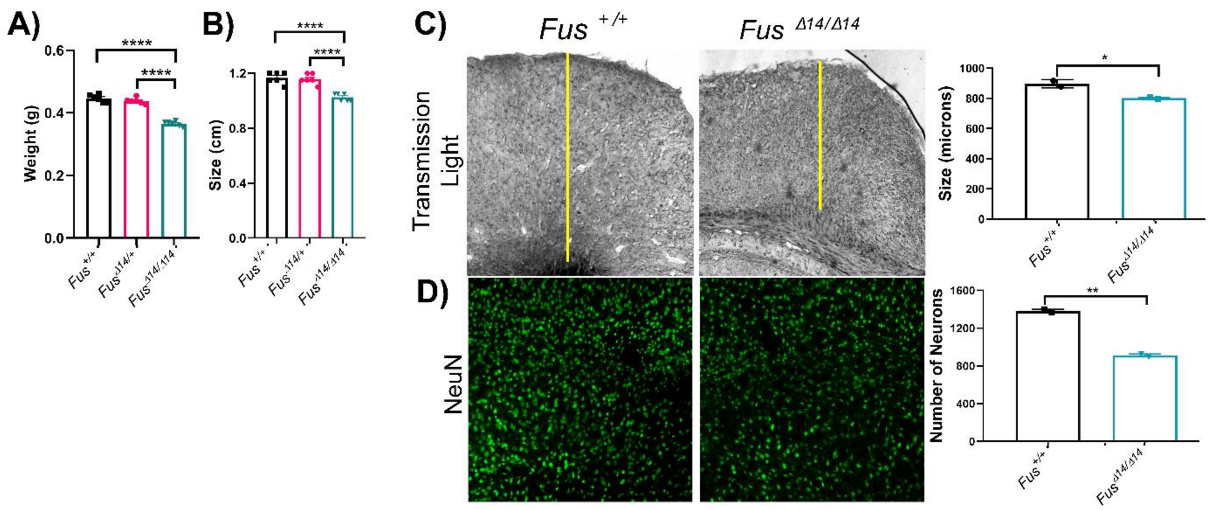

Fig. S4. Male brain morphology and structure. Brain weight (**A**) and size (**B**) measurements from male mice perfused at 9-10 weeks of age (n= 6 per group). **C.** Representative light transmitted images showing histological coronal sections of the frontal cortex of male *Fus*$^{+/+}$ (n = 2) and *Fus*$^{\Delta14/\Delta14}$ (n = 2) male mice at 10 weeks of age. Yellow lines represent the total length of the cortex. Scale bar = 100 µm. Graph shows the quantification of cortex thickness comparing the two groups. **D.** Representative confocal images showing staining of histological sections with the nuclear neuronal marker NeuN (green). Scale bar = 50 µm. The graph shows quantification of the total number of NeuN + cells per area of the cortex. The data represents the average number of 3 areas analysed per mouse, same as C. Data was analysed using the unpaired *t*-test.

Disease Models & Mechanisms • Supplementary information

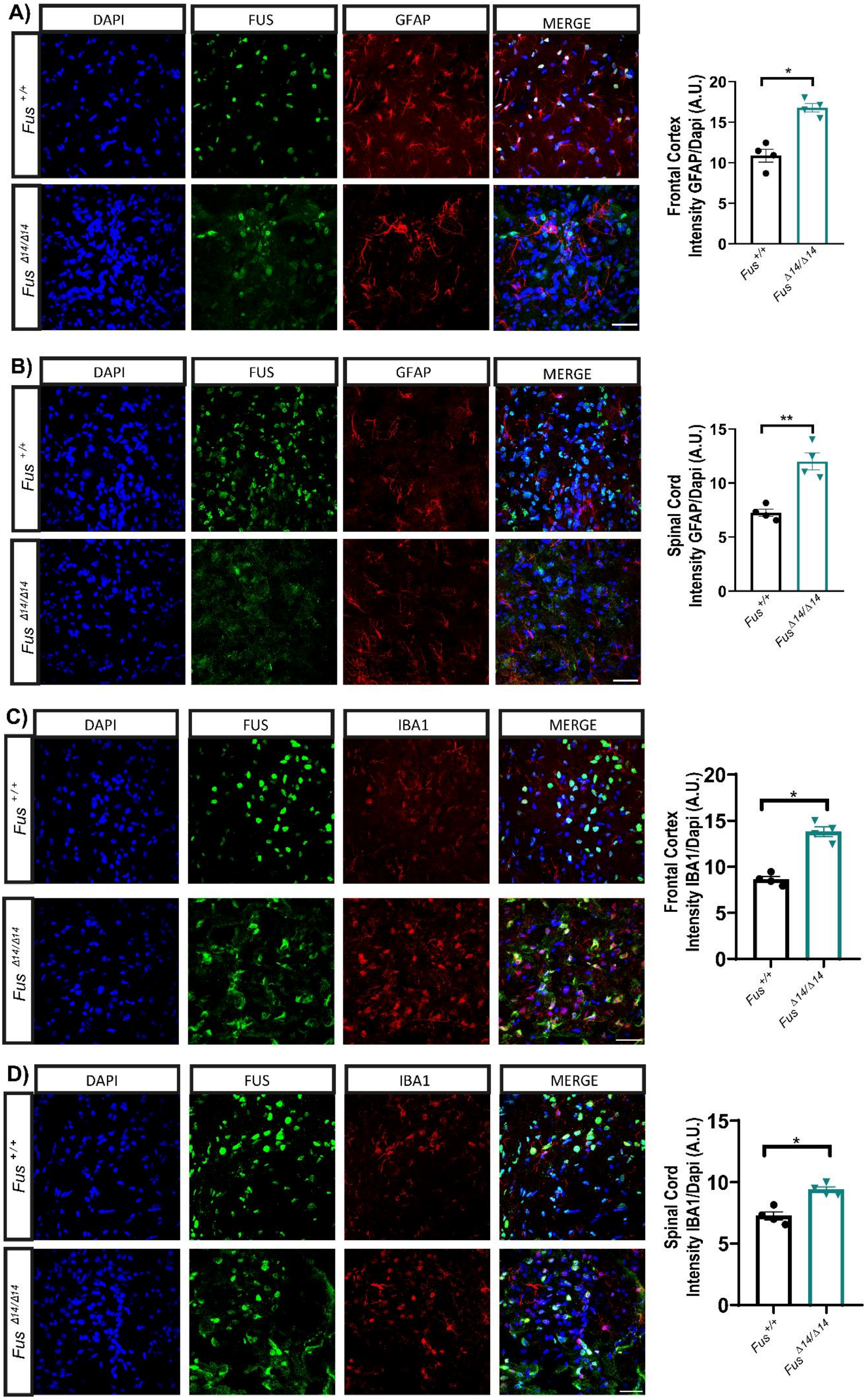
A)
DAPI
FUS
GFAP
MERGE
Fus +/+
Fus Δ14/Δ14
Frontal Cortex
Intensity GFAP/Dapi (A.U.)
*
B)
DAPI
FUS
GFAP
MERGE
Fus +/+
Fus Δ14/Δ14
Spinal Cord
Intensity GFAP/Dapi (A.U.)
**
C)
DAPI
FUS
IBA1
MERGE
Fus +/+
Fus Δ14/Δ14
Frontal Cortex
Intensity IBA1/Dapi (A.U.)
*
D)
DAPI
FUS
IBA1
MERGE
Fus +/+
Fus Δ14/Δ14
Spinal Cord
Intensity IBA1/Dapi (A.U.)
*

Fig. S5. GFAP and Iba1 + staining on frontal cortex and spinal cord from FUS+/+ and FUSΔ14/Δ14 mice. Representative confocal images showing FUS staining (in green), GFAP staining (in red), nuclei (stained with DAPI, in blue) and a merger of the three images, from frontal cortex **(A)** and spinal cord **(B).** Histological sections of perfused *Fus*$^{+/+}$ (n = 3) and *Fus*$^{\Delta14/\Delta14}$ (n = 4) female mice at 10 weeks of age. The graph shows the quantification of the total GFAP intensity in relation to the number of cells (by DAPI) per area. Representative confocal images showing FUS staining (in green), Iba1 staining (in red), nuclei (stained with DAPI, in blue) and a merger of the three images, from frontal cortex **(C)** and spinal cord **(D)** histological sections of perfused *Fus*$^{+/+}$ (n = 3) and *Fus*$^{\Delta14/\Delta14}$ (n = 4) female mice at 10 weeks of age. The graph shows the quantification of the total Iba1 intensity in relation to the number of cells per area. Please note that the nuclear staining of IBA1, in red, is non-specific. Scale bar = 20 µm. FC= Frontal cortex; SPC= Spinal Cord. Data is represented as the mean ± SEM and analysed using unpaired t-test. *p< 0.05, **p < 0.01.

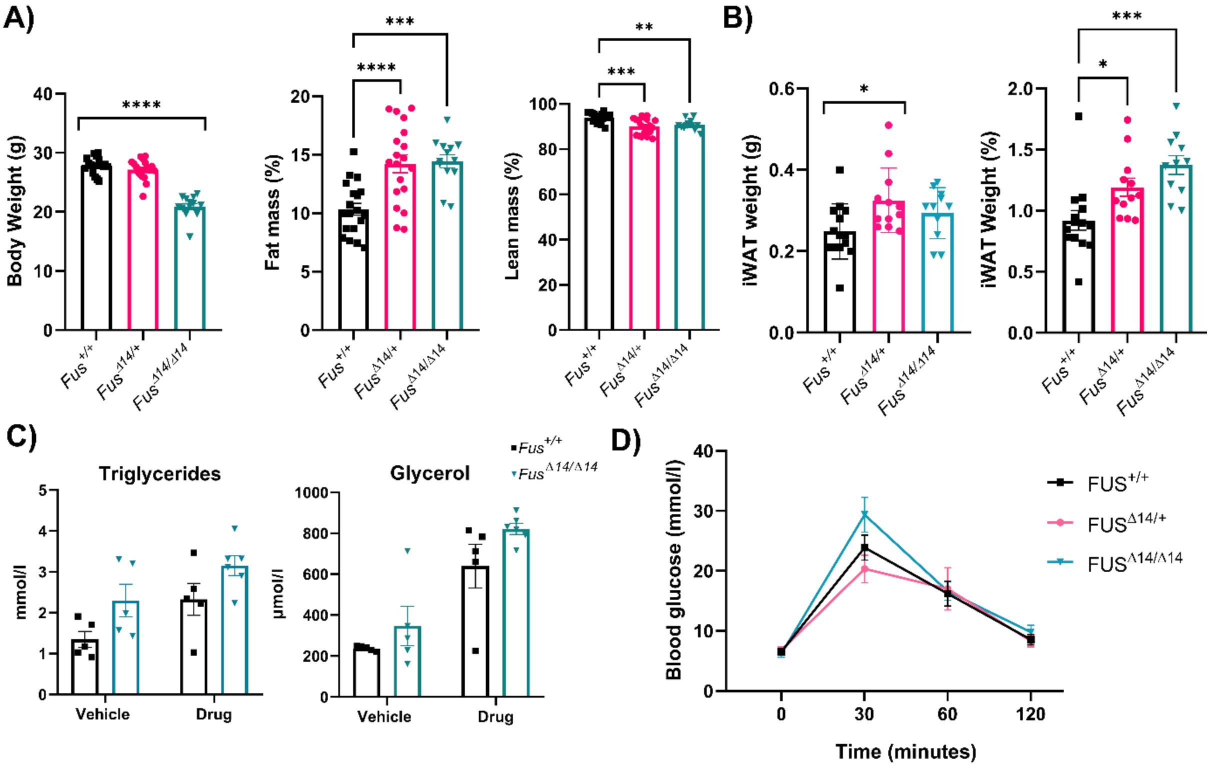

Fig. S6. Additional metabolic characterisation. A. Body composition analysis by EchoMRI scans in 10-week old female mice. Fat and lean mass is presented as the percentage of the total body weight. *Fus*$^{\Delta14/+}$ and *Fus*$^{\Delta14/\Delta14}$ mice have more corrected fat mass compared to *Fus*$^{+/+}$ littermate mice. Males: n = 27 *Fus*$^{+/+}$, n = 22 *Fus*$^{\Delta14/+}$, n = 17 *Fus*$^{\Delta14/\Delta14}$, **B.** Dissected iWAT weights from 10-week old female mice. *Fus*$^{\Delta14/+}$ and *Fus*$^{\Delta14/\Delta14}$ mice have bigger iWAT depots proportionally to their total body weight when compared to wild-types littermates. Data shown as mean ± SEM and analysed using the one-way ANOVA followed by Dunnett's multiple comparisons test. N = 6 mice per group. **C.** Graph showing measures of serum triglycerides and glycerol in basal conditions and after the lipolysis induction in male mice at 9-weeks of age. n = 5 mice per group. **D.** Intraperitoneal glucose tolerance test (IPGTT) in female mice at 9-weeks of age. Data analysed using 2-way ANOVA followed by Tukey multiple comparisons test. N = 9 per group. $^{**}p < 0.01$, $^{***}p < 0.001$.

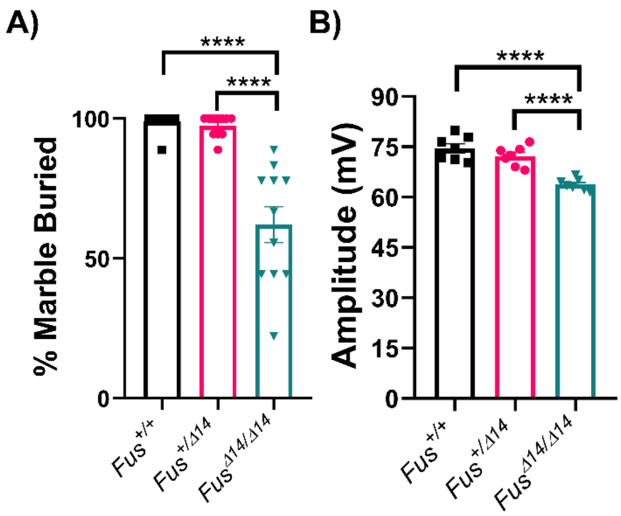

Fig. S7. Cognitive and motor tests in female mice. A. Marble burying test. The percentage of marbles two-thirds buried was recorded. (n=11 mice per group). **B.** EMG test. CMAP amplitude in the hind-limbs at 9-weeks: n = 11 $Fus^{+/+}$, n = 12 $Fus^{\Delta14/+}$and n = 12 $Fus^{\Delta14/\Delta14}$. Data analysed using One-way ANOVA. Data in graphs represents the mean ± SEM. **** $p< 0.0001$.

Disease Models & Mechanisms • Supplementary information

Table S1. List of DEGs in all tissues.

Click here to download Table S1

Table S2. List of primers used.

Primer Name	Sequence 5´→3´	
FUS Forward	GGTTGGGAGAATGGAGCTGA	
FUS Reverse	GATTAGGAGGTGGGCTAGGG	
**Primer Name**	**ASSAY ID**	**SUPPLIER**
Adiponectin (Adipoq)	Mm00456425_m1	ThermoFisher Scientific
Peripilin 1 (Plin1)	Mm00558672_m1	ThermoFisher Scientific
Glucose Transporter 4 (Glut4)	Mm00436615_m1	ThermoFisher Scientific
Peroxisome proliferator activated receptor gamma (Pparγ)	Mm01184322_m1	ThermoFisher Scientific
Fatty acid synthase (FASN)	Mm00662319_m1	ThermoFisher Scientific
Lipase (Lipe)	Mm00495359_m1	ThermoFisher Scientific
Calnexin (Canx)	Mm00500330_m1	ThermoFisher Scientific

Disease Models & Mechanisms • Supplementary information

Table S3. List of antibody and reagents used.

PRODUCT	SUPPLIER	REFERENCE	DILUTIONS
**Phire Tissue Direct PCR Master Mix**	ThermoFisher Scientific	F170S	
**Agarose**	ThermoFisher Scientific	R0492	
**TBE Buffer, Tris-Borate-EDTA, 10X Solution, Electrophoresis**	Fisher BioReagents™	BP1333-1	
**MIDORI Green Xtra**	Nippongenetics	MG10	
**D-Digit**	Licor	DDG-000147	
**Grip Meter**	Bioseb	BIO-GS3	
**Lidocaine/Prilocaine**	Aspen Pharmacare		
**Accu-Chek® Aviva**	Accu-Chek®		
**Accu-Chek® strips**	Accu-Chek®		
**Lipolysis test**	Cayman Chemical	CL316243	
**Chemistry Analyser**	Beckman Coulter	AU680	
**Fentanest**	KernPharma	756650.2H	
**Thiobarbital**	Braun		
**PFA without methanol**	Quimipur	E/AA6/J-1276	
**PBS, Phosphate Buffered Saline, 10X Solution**	Fisher BioReagents™	BP3994	
**Paraffin**	Roth	6642.2	
**OCT**	ThermoFisher Scientific	6502	
**CoverSlip**	Knittel	100268	
**Oil Red O**	Merck-Sigma	O0625-25G	
**Cryostat**	Leyca	CM1950	
**FUS Antibody**	Novus	NB100-565	1/300
**HOESCHT**	ThermoFisher Scientific	H21492	1/2000
**GFAP Antibody**	Cell Signaling	36565	1/300
**Iba1 Antibody**	Abcam	ab5076	1/200
**NeuN Antibody**	Cell Signaling	54761s	1/200
**Alexa 488 rabbit**	Invitrogen	A32731	1/1500
**Alexa 488 mouse**	Invitrogen	A32723	1/1500
**Alexa 594 rabbit**	Invitrogen	A11037	1/1500
**Alexa 594 mouse**	Invitrogen	A11032	1/1500
**Alexa 647**	Invitrogen	A32733	1/1500
**Gadolinium**	Gadovist		
**RNeasy Lipid Tissue Mini Kit**	Qiagen	74804	
**cDNA Reverse Transcriptase Kit**	ThermoFisher Scientific	A45003	
**Fast Sybr Green Mastermix**	ThermoFisher Scientific	4385612	
**RNA Nano 6000 Kit**	Agilent Technology		

**NEBNext Ultra™ RNA Library Prep Kit**	Illumina	NEB#E7770	
**Ripa-Buffer**	ThermoFisher Scientific	89900	
**Centrifuge 5424R**	Eppendorf		
**Protease Inhibitor Cocktail Tablets**	Roche	04693116001	
**Phosphatase Inhibitor Cocktail Tablets**	Roche	04906837001	
**BSA 10 mg/ml**	New Englands Biolab	174P0753S	
**DC Protein Assay Reagent S**	BioRad	#500-115	
**DC Protein Assay Reagent A**	BioRad	#500-113	
**DC Protein Assay Reagent B**	BioRad	#500-114	
**Tween 20**	Fisher BioReagents™		
**Protein Stain Sample Pack**	Licor	#DO1103-03	
**MOPS Running Buffer**	Invitrogen Novex	NP001	
**MES Running Buffer**	Invitrogen Novex	NP002	
**LDS Sample Buffer**	Invitrogen	NP007	
**PVDF membrane**	Immobilion	IPFL00010	
**Whatman Filter**	Whatman	10427806	
**SurePage, Bis-Tris 10%, 15 wells**	GeneScrip	M00666	
**Donkey Anti-Goat 555**	Abcam	Ab150134	1/1500
